# Pharmacological signatures of the reduced incidence and the progression of cognitive decline in ageing populations suggest the protective role of beneficial polypharmacy

**DOI:** 10.1371/journal.pone.0224315

**Published:** 2019-11-06

**Authors:** Anatoly L. Mayburd, Mathilda Koivogui, Ancha Baranova

**Affiliations:** 1 Neurocombinatorix, Alexandria, Virginia, United States of America; 2 George Mason University, School of Systems Biology, Colgan Hall, MSN 3E1 George Mason University, Manassas, Virginia, United States of America; 3 Neurocombinatorix, Alexandria, Virginia, United States of America; 4 Research Centre for Medical Genetics, Moscow, Russia; Memorial Sloan-Kettering Cancer Center, UNITED STATES

## Abstract

Preventive treatments for dementia are warranted. Here we show that utilization of certain combinations of prescription medications and supplements correlates with reduced rates of cognitive decline. More than 1,900 FDA-approved agents and supplements were collapsed into 53 mechanism-based groups and traced in electronic medical records (EMRs) for >50,000 patients. These mechanistic groups were aligned with the data presented in more than 300 clinical trials, then regression model was built to fit the signals from EMRs to clinical trial performance. While EMR signals of each single agents correlated with clinical performance relatively weakly, the signals produced by combinations of active compounds were highly correlated with the clinical trial performance (R = 0.93, p = 3.8 x10^-8). Higher ranking pharmacological modalities were traced in patient profiles as their combinations, producing protective complexity estimates reflecting degrees of exposure to beneficial polypharmacy. For each age strata, the higher was the protective complexity score, the lower was the prevalence of dementia, with maximized life-long effects for the highest regression score /diversity compositions. The connection was less strong in individuals already diagnosed with cognitive impairment. Confounder analysis confirmed an independent effect of protective complexity in multivariate context. A sub-cohort with lifelong odds of dementia decreased > 5-folds was identified; this sub-cohort should be studied in further details, including controlled clinical trials. In short, our study systematically explored combinatorial preventive treatment regimens for age-associated multi-morbidity, with an emphasis on neurodegeneration, and provided extensive evidence for their feasibility.

## Introduction

Alzheimer’s disease currently affects 5 million Americans; by 2050, this number is expected to grow to 16 million [1, 2]. Currently, about 10% of Americans after 65 live with dementia [1, 3]. Recent decreases in the rate of dementia and a shift to later ages of onset are encouraging [4]. However, in a first quartile of life expectancy, dementia still accounts for 1 in every 6 deaths, and is a major source of disability [1]. While multiple clinical trials for single dementia combating agents are ongoing [5], earlier stages of neurodegeneration process largely remain unexplored in humans.

On the other hand, recent studies in model animals are encouraging. Particular successful efforts in visible rejuvenation of already differentiated tissues are reviewed in [6]). NAD+ supplementation has been associated with an extension of murine lifespan by 10–15% [7, 8] through the mechanism likely related to overall brain function. The maintenance of microvasculature [9, 10], the clearance of misfolded proteins [11], the neuroprotection [12, 13], and the regulation of cholinesterase [13] were shown to be comparably important for the prevention of neurodegeneration in various models. Moreover, human TRIIM (Thymus Regeneration, Immunorestoration, and Insulin Mitigation) trial brought evidence that a combination of recombinant human growth hormone, DHEA and metformin produces a regression of multiple aspects and biomarkers of aging in treated men [14]. No successful combinatorial treatments aimed at reversing neurodegeneration were, however, reported in humans so far.

Given that the neurodegenerative diseases proceed along a variety of detrimental pathophysiological pathways, which are additive but not necessarily related to each other, and that the full extent of neurodegeneration takes a substantial time to develop, testing of active agent ensembles may represent a promising strategy for combating neurodegeneration. Therefore, repurposing of known effective medication already approved for human use as components of anti-dementia drug cocktails may be a relatively inexpensive approach for delaying progression of Alzheimer’s and other types of age-associated neurodegeneration.

In this report we have focused on discovering possible combinatorial pharmacological means to prevent dementia, or, at the very least, to delay its onset/ or slow rate of its progression [5]. Here we present epidemiological patterns we have observed in several databases of electronic medical records reporting potentially beneficial polypharmacy. Presented analysis attempts to avoid single theoretical assumption bias by data-driven exploration of an evidence of incremental success observed in multiple clinical trials. Since the effects of standalone agents are likely small, each of them is not likely to show any translational value when tested in controlled clinical trials individually [15, 16]. On the other hand, when applied simultaneously, these agents may engage multiple molecular mechanisms, or differentially impact partially overlapping cohorts of susceptible patients, thus, providing larger overall effect in a general population of patients. Thus, cumulative effect of beneficial polypharmacy may exceed one predicted by a purely additive model. The prototype components of such efficient cocktails may be mined by retrospective analysis of exposures reflected in diverse data sources, or, at the very least, may help to identify exposure-dependent sub-cohorts with reduced rates of dementia.

To advance the development of novel therapies for Alzheimer’s disease, we performed statistical analysis of the effects of individual agents, then show that patients’ groups exposed to certain combinatorial treatments have their rates of diagnosing dementia dramatically reduced. The data on individual drugs were further mined by meta-analysis of evidence resulting from randomized placebo-controlled trials as well as preclinical research, and, for cross-validation, aligned with database-driven leads. Validated leads were traced in the patient’s profiles as groups, and these groups were explored for hazard ratios of dementia reduction and relative influence of other factors. The practical application of this effort is nomination of relatively novel principles that can be tested in controlled clinical trials.

## Methods

### Data sources

**Fig 1:**
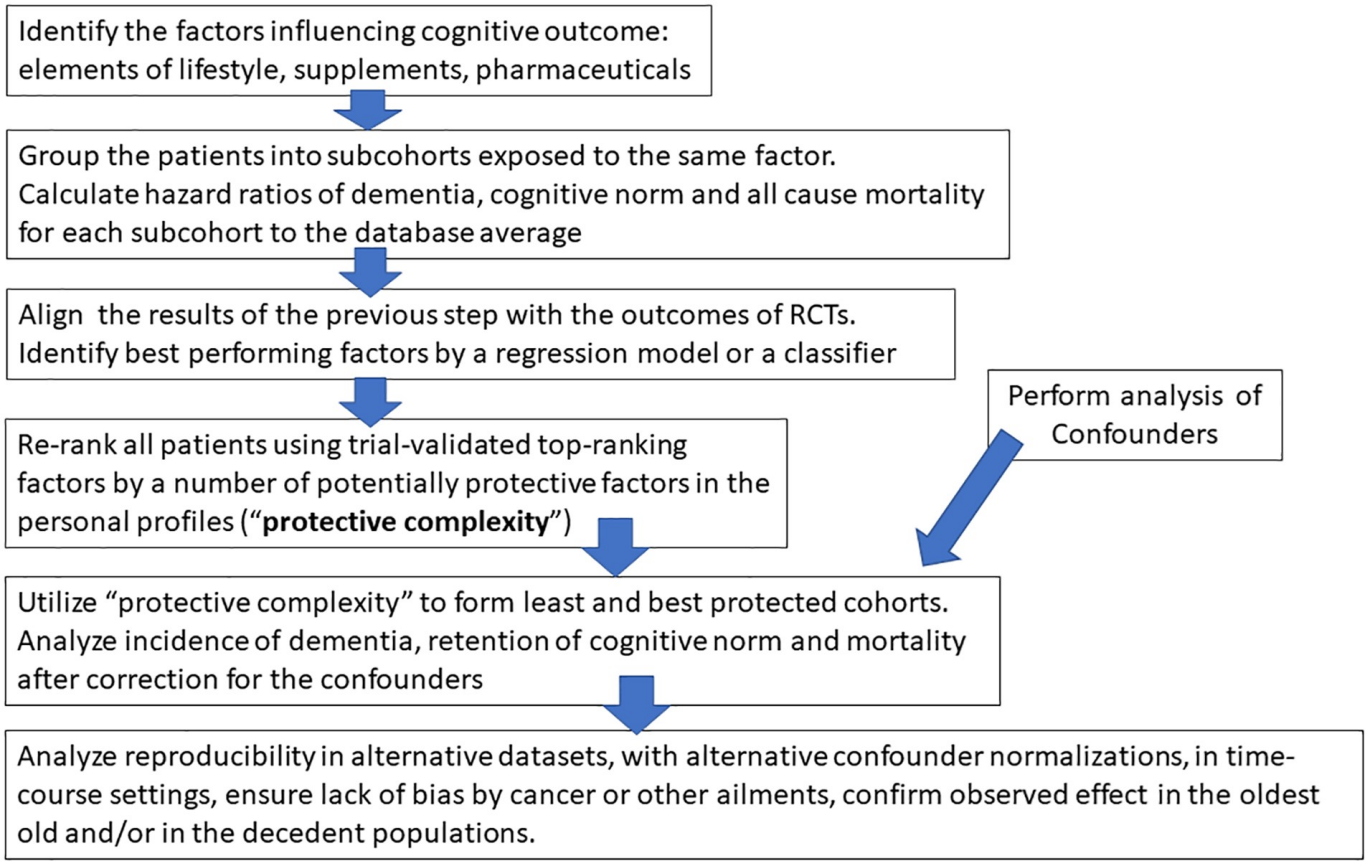
Computational screening of combined prescription drugs, supplements and lifestyle factors as potential multi-factor modifiers of dementia phenotypes.

**Fig 2:**
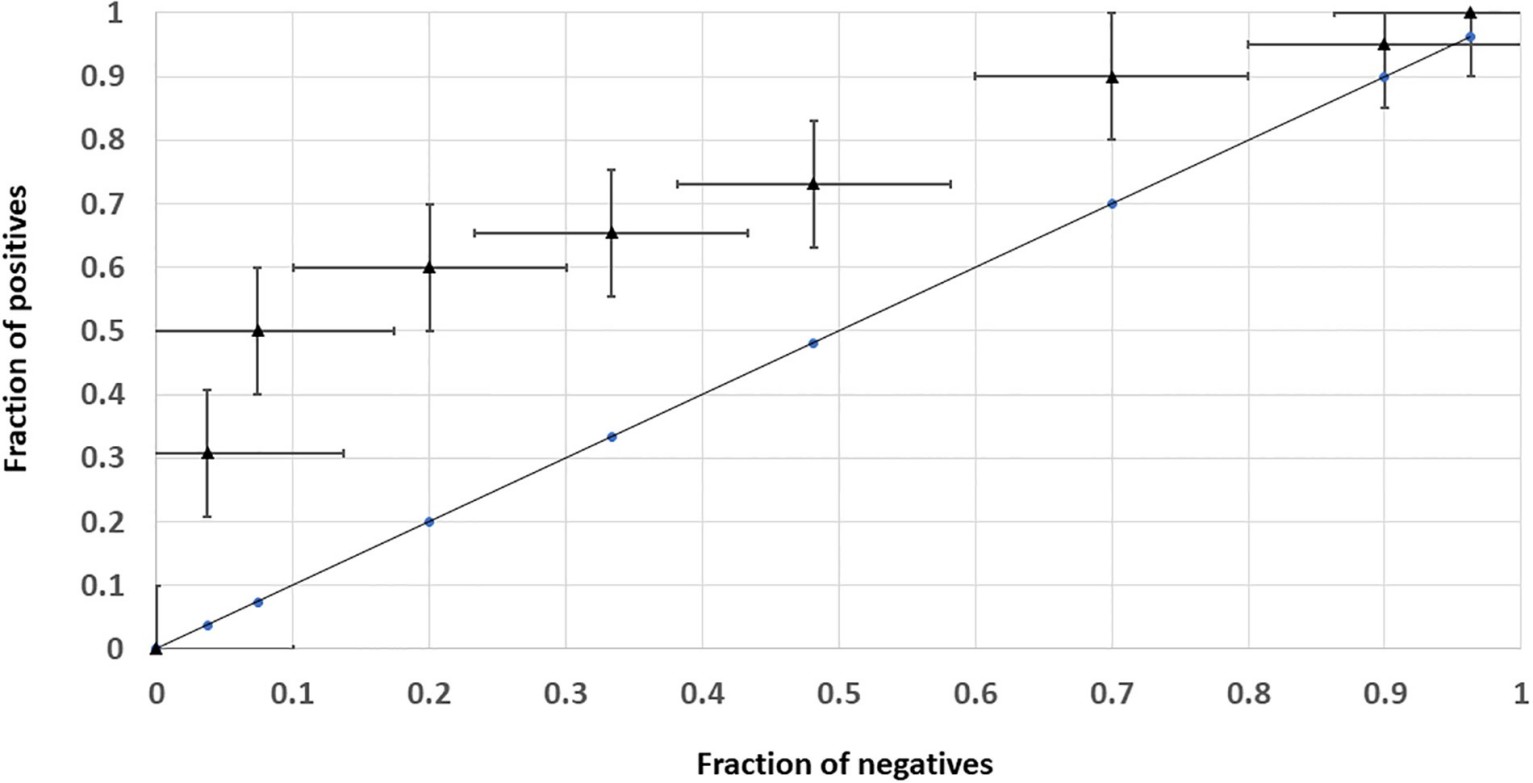
Receiving operating characteristics for classifier REG. The negatives are BAL/TOT ratios < 0, the positives are BAL/TOT ratios > 0. The ROC curve was constructed by ranking the mechanistic aggregates of agents with the negative BAL/TOT according to REG, dividing the rank in 10% intervals and counting the fractions of positives in each 10% interval. At each point of the plot, the cumulative fraction of negatives and positives forms the coordinate. At the minimal position of the rank by REG, the cumulative coordinate is (1.0, 1.0) in terms of both negatives and positives. The uncertainty of the point’s position marked by vertical and horizontal bars results from variation in the prediction rule observed at 5:1 cross-validation described earlier.

**Fig 3:**
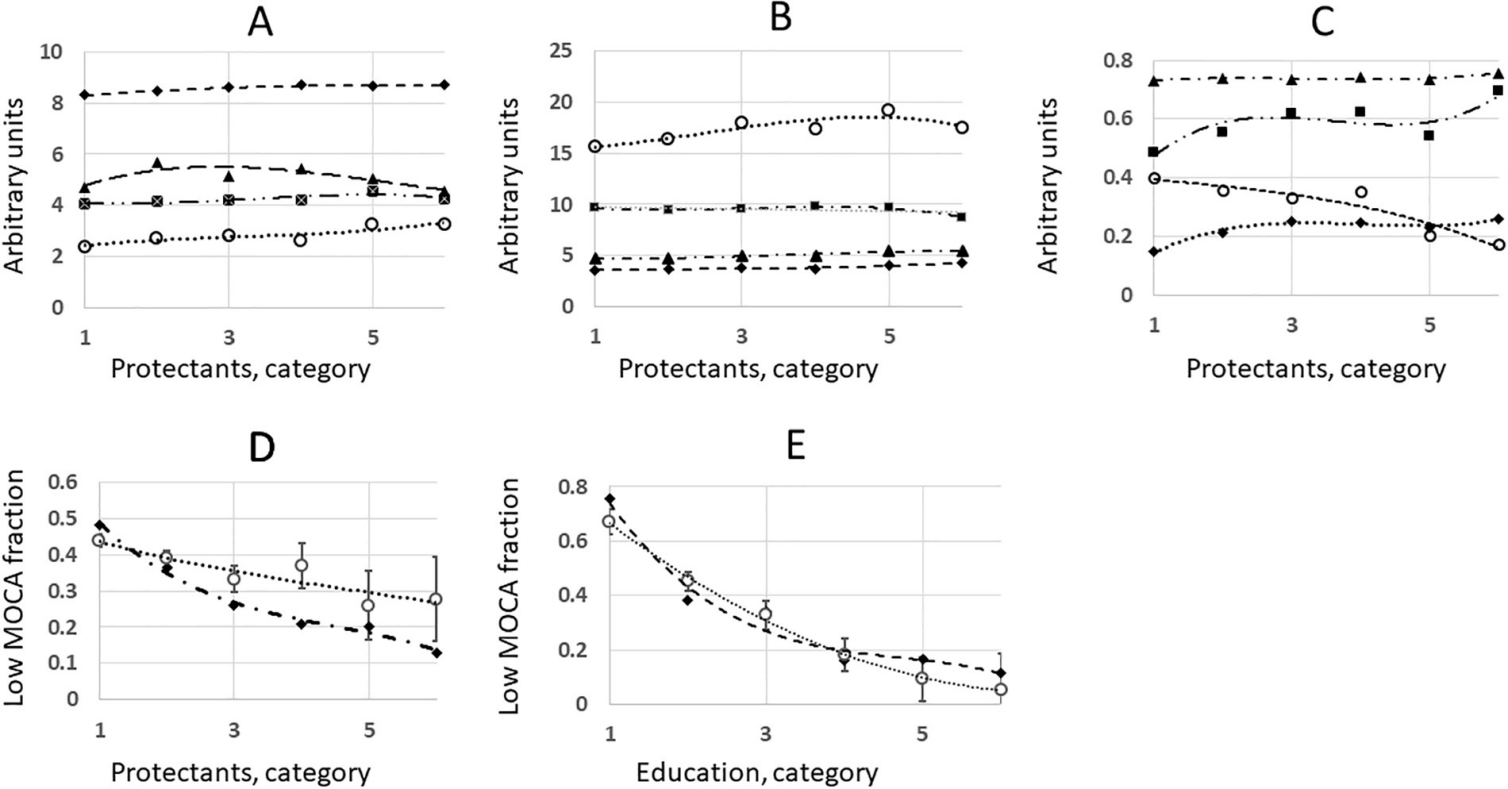
Potential confounders of the protective complexity in NSHAP dataset (2,138 respondents). The number of protectants in a personal profile was categorized as 1–0, 1; 2–2, 3; 3–4, 5; 4–6, 7; 5–8, 9; 6–10, 11. Education was categorized as 1 –elementary school; 2 –middle school; 3 –high school; 4–2 years of college; 5–4 years of college; 6 –advanced or professional degree. The factors traced were: A. Diamond symbols–vision and hearing; triangles–number of prescription medicines; crossed circles–income; hollow circles–education. B. Hollow circle symbols–happy, confident or relaxed mood; squares–number of hours spent sleeping; triangles–self-assessed physical health; diamonds–self-assessed mental health. C. Triangle symbols–age/100; squares–social consumption of alcohol; hollow circles–errors in cognitive tests administered at baseline; diamonds–osteoarthritis. D. Hollow circle symbol–background model of low MOCA fractions, linking all confounders in the same multivariate context; diamonds–measured values of low MOCA fractions; CI95 confidence intervals are provided. The low MOCA fractions are plotted as a function of protective complexity category. E. Hollow circle symbol–background model of low MOCA fractions; diamonds–measured values of low MOCA. The low MOCA fractions are plotted as a function of education category.

**Fig 4:**
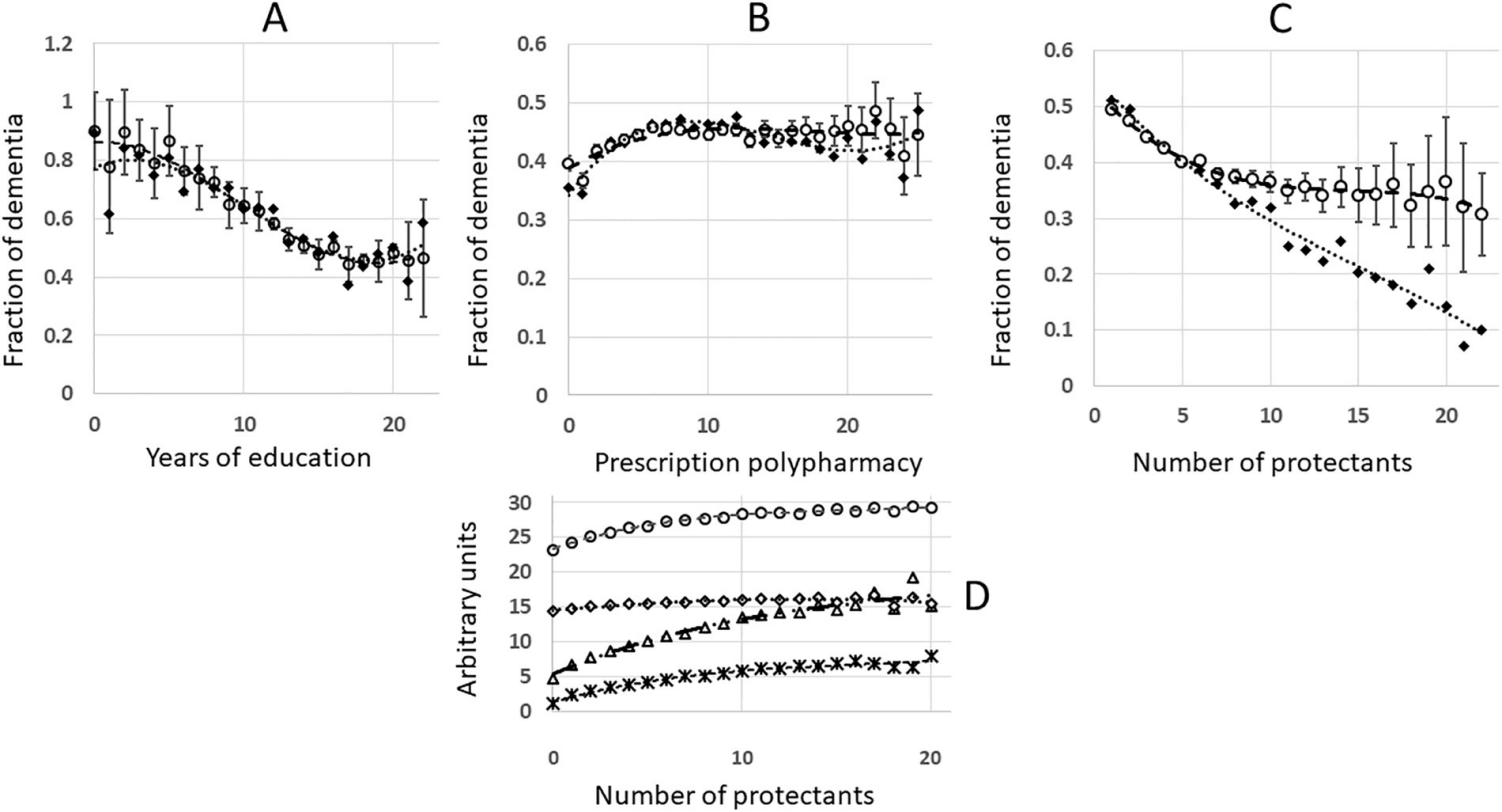
Background models constructed by combining potential confounders. A. Testing background model in NACC (38837 patients). Fraction of dementia at the end of follow-up was plotted as a function of educational achievement, years. Hollow circles symbolize the background model, combining all confounders. Diamonds symbolize observed values. B. Testing background model in NACC (38837 patients). Fraction of dementia at the end of follow up is plotted as a function of prescription polypharmacy actives per a person. Hollow circles symbolize the background model, combining all confounders. Diamonds symbolize observed values. C. Plotting of dementia at the end of follow up as a function of protectant number in NACC. Hollow circles symbolise the background model combining all confounders in a multivariate context, diamonds symbolize the observed dementia values. D. Distribution of potential confounders as a function of protective complexity in NACC. Hollow circles symbolize MMSE cognitive score at baseline, diamonds–years of education, triangles–polypharmacy at baseline, number of prescriptions, star symbol–length of follow up, years.

The NACC database is affiliated with National Alzheimer’s Coordination Center [17]. This source has collected multi-centre data nation-wide since 2007, and the release as of 05/2017 was analysed for Tables 1–4, Figs 1 and 2. For this release, all versions (1–3) were accepted in the analysis. The database of this release is organized longitudinally with 118,200 visits for 35,200 de-identified patients, and includes comorbidities, ages of entering and exiting observation, cognitive status, family and demographic data, alive/dead flag and pharmaceuticals taken during each visit. The dosages are not provided by NACC and were assumed to be typical prescription dosages. A newer release of 09/2018 was used for Tables 5 and 6, Figs 3 and 4.

**Table 1:** Functional clusters of known pharmaceuticals aligned with the integrated data from NACC as well as randomized clinical trials (RCTs).

**Pharmacological groups tested in RCTs**										
**Mechanistic Category**	**RCT+**	**RCT-**	**RCT 0**	**MOD**	**BAL/TOT**	**TOT**	**HRDEM**	**HRAMRT**	**HRNORM**	**REG**
**Antimigraine**	**1**	**0**	**0**	**2**	**1**	**1**	**0.52**	**0.25**	**1.44**	**2.04**
**Vitamin A**	**1**	**0**	**0**	**5**	**1**	**1**	**0.69**	**0.74**	**1.29**	**1.95**
**Oestrogen**	**4**	**2**	**3**	**1.22**	**0.22**	**9**	**0.49**	**0.48**	**1.53**	**1.93**
**Selenium**	**2**	**1**	**0**	**3.67**	**0.33**	**3**	**0.59**	**0.62**	**1.34**	**1.9**
**Chromium**	**1**	**0**	**0**	**1**	**1**	**1**	**0.48**	**0.42**	**1.5**	**1.89**
**Zinc**	**1**	**0**	**0**	**5**	**1**	**1**	**0.76**	**0.81**	**1.13**	**1.73**
**Supplements**	**23**	**3**	**4**	**3.4**	**0.66**	**30**	**0.65**	**0.57**	**1.21**	**1.64**
**Multivitamins**	**2**	**2**	**1**	**5**	**0**	**5**	**0.88**	**0.79**	**1.11**	**1.62**
**Herbal**	**14**	**1**	**1**	**2.56**	**0.81**	**16**	**0.77**	**0.48**	**1.33**	**1.56**
Antioxidant	10	6	1	2.28	0.23	17	0.66	0.56	1.25	1.53
Vitamin C	2	2	0	3.5	0	4	0.83	0.76	1.16	1.5
**Immunomodulators**	**21**	**1**	**3**	**1.24**	**0.8**	**25**	**0.61**	**0.85**	**1.24**	**1.46**
Calcium	0	1	0	2	-1	1	0.74	0.65	1.24	1.43
PDE5	0	1	0	1	-1	1	0.52	0.49	1.19	1.41
Spironolactone	1	0	0	1	1	1	0.74	1.44	1.14	1.37
S-adenosylmethionine	1	0	0	1	1	1	0.74	1.44	1.14	1.37
Naproxen	2	2	0	1.5	0	4	0.68	0.62	1.22	1.38
Anticancer	0	0	1	1	0	1	0.69	0.92	1.22	1.37
Antivirals	2	0	0	1	1	2	0.56	0.66	1.17	1.36
Vitamin B	4	1	1	4.33	0.5	6	1.07	0.92	0.99	1.33
Vitamin B6	4	6	4	3	-0.14	14	0.83	0.75	1.06	1.32
Omega-3	15	7	3	1.44	0.32	25	0.74	0.58	1.2	1.29
Oestrogen mimic	1	1	0	1	0	2	0.76	0.71	1.22	1.27
Celecoxib	1	2	0	1.33	0.33	3	0.76	0.9	1.12	1.25
Ibuprofen	0	1	0	1	-1	1	0.71	0.55	1.21	1.27
Antihistamine	1	3	1	1	-0.4	5	0.76	0.79	1.18	1.25
**Magnesium**	**3**	**0**	**0**	**1**	**1**	**3**	**0.75**	**0.83**	**1.15**	**1.23**
Folate	9	4	4	3.06	0.29	17	0.94	0.98	0.96	1.23
Coenzyme Q10	1	3	0	1.25	-0.5	4	0.74	0.46	1.18	1.21
Vitamin E	6	6	0	3.08	0	12	1.03	0.94	0.98	1.19
NSAIDs	0	0	1	1	0	1	0.79	0.89	1.09	1.15
Antibiotics	1	6	0	1.75	-0.71	7	1.06	1.39	0.98	1.15
Hormonal	6	2	2	1.1	0.4	10	0.83	0.72	1.13	1.14
Antiplatelet	0	1	1	1.5	-0.5	2	0.98	1.25	0.96	1.09
**Vitamin D**	**1**	**1**	**2**	**1.25**	**0**	**4**	**0.78**	**0.36**	**1.18**	**1.12**
Gingko	2	4	2	5	-0.25	8	1.3	0.86	0.85	1.12
Analgesic	1	0	0	1	1	1	1	1.39	0.96	1.06
Antihypertensive	7	8	1	1.06	-0.06	16	0.93	1.03	1.01	1.04
Vitamin B12	8	7	4	3.16	0.05	19	1.06	0.82	0.91	1.06
Omega-6	0	1	0	1	-1	1	1	0.83	1.05	0.96
Anti-ALS	0	1	0	1	-1	1	1.06	0.83	1.05	0.93
**Metformin**	**2**	**0**	**0**	**1**	**1**	**2**	**0.92**	**0.75**	**0.99**	**0.92**
Antidiabetic (not insulin or metformin)	0	2	0	1.5	-1	2	1.09	1.04	0.89	0.91
Stimulants	4	2	2	1.13	0.25	8	0.96	0.89	0.91	0.89
Statins	1	3	1	1	-0.4	5	1.03	0.92	0.94	0.87
Overactive bladder agents	0	2	0	2	-1	2	1.32	1.26	0.76	0.85
Fluoxetine	1	0	0	1	1	1	1.18	1.14	0.86	0.81
Levodopa	0	0	1	1	0	1	1.67	1.74	0.38	0.88
Anticonvulsant	0	1	0	1	-1	1	1	1.57	0.48	0.81
Insulin	3	0	0	1	1	3	1.09	1.01	0.86	0.8
Anti-Parkinson’s	2	0	1	1	0.66	3	1.22	1.22	0.72	0.73
Antidepressant	0	4	0	1	-1	4	1.46	1.28	0.57	0.61
Lithium	2	1	0	1	0.33	3	1.25	0.98	0.5	0.52
**Pharmacological groups tested in clinical studies and in animal experiments but not in RCTs**										
**Mechanistic category**			**HRDEM**		**HRAMRT**		**Supporting literature**			
Antiprotozoan			0.71		0.93		[24]			
Bronchodilator			0.84		1.02		[24], [25], [36]			
**Biotin**			**0.21**		**0.09**		[26], [27]			
Adrenaline			0.44		0.25		[28]			
Antidiabetic			1.1		1.17		[29]			
Chondroitin / glucosamine			0.62		0.5		[30]			
Antiglaucoma			0.86		1.13		[31]			
Probiotic			0.7		0.5		[32]			
Anti-gout			0.97		1.1		[33], [34]			
Corticosteroid			0.84		1.06		[35], [37]			

HR DEM–hazard ratio of all-cause dementia in the category of compounds aggregated by mechanism of action, HR AMRT–hazard ratio of all-cause mortality, HR NORM–hazard ratio of the patients remaining cognitively normal in the aggregate. The hazard ratios were computed by dividing the value observed in the mechanism aggregate to the value observed across the entire database.

RCT +, RCT -, RCT 0 –numbers of randomized clinical trials (RCTs) directed to the prophylaxis and treatment of neurodegeneration with positive, negative and neutral outcomes, respectively. The trials are attributed to the pharmacological mechanisms or aggregates of the latter. A positive outcome was defined as confirmation of an original hypothesis, a negative outcome was defined as non-confirmation, and a neutral outcome was defined as inconclusive. (RCT+—RCT-)/TOT is the ratio between the positive balance of clinical trials and total trial outcomes.

MOD–modality; the average number of ligands tested in the trials of the mechanistic aggregate.

REG–regression model combining cognitive metrics and mortality for a mechanism (see Results).

Constituents of certain broader categories were as follows: “Immunomodulators” (zinc, natalizumab, interferon β-1a, rituximab, peginterferon, oral delayed-release dimethyl fumarate, ocrelizum, laquinimod, human polyclonal IgG antibody (IVIG), glatiramer, fingolimod, everolimus, amiselimod); “Supplements” (L-cysteine, cerebrolysin, actovegin, phosphatidylserine, phosphatidic acid, creatinine, choline, carnosine, anserine, taurine, inositol, homotaurine, D-serine, choline cytidine diphosphate, acetyl-L-carnitine, S-adenosylmethionine, N-acetyl cysteine, and L-theanine); “Herbals” (Korean multi-herb mixtures, rosemary, resveratrol, green oats extract, *Bacopa monnier*i, green tea, turmeric, saffron, and curcumin); “Antioxidants” (complex antioxidant blends, -β-carotene, anthocyanin, cherry juice, flavone-rich orange juice, α-lipoic acid, lutein, zeaxanthin, macular carotenoids); “Hormonal group” (leuprolide acetate, pregnenolone, growth hormone-releasing hormone, dehydroepiandrosterone (DHEA), melatonin (extended release) and testosterone).

The agents demonstrating a promise are highlighted in bold based on REG score, incremental success of clinical trials, or distributions of REG for individual dosage forms between the highest and the remaining octiles of the rank (see below, text).

**Table 2:** Testing set for assessing performance of the supplements and pharmaceuticals with the maximal REG value (the top octile of the rank in Table 1).

Source	Agent	MOD	S
Vuralli D, Ayata C, Bolay H. Cognitive dysfunction and migraine. J Headache Pain. 2018 Nov 15;19(1):109.	Antimigraine	1	N/A
Veasey RC et al. The Effects of Supplementation with a Vitamin and Mineral Complex with Guaraná Prior to Fasted Exercise on Affect, Exertion, Cognitive Performance, and Substrate Metabolism: A Randomized Controlled Trial. Nutrients. 2015 Jul 27;7(8):6109–27.	Biotin	5	+1
Sedel F et al. High doses of biotin in chronic progressive multiple sclerosis: a pilot study. Mult Scler Relat Disord. 2015 Mar;4(2):159–69	Biotin	1	+1
Tourbah A et al. MD1003 (high-dose biotin) for the treatment of progressive multiple sclerosis: A randomised, double-blind, placebo-controlled study. Mult Scler. 2016 Nov;22(13):1719–1731	Biotin	1	+1
Tourbah A et al. MD1003 (High-Dose Pharmaceutical-Grade Biotin) for the Treatment of Chronic Visual Loss Related to Optic Neuritis in Multiple Sclerosis: A Randomized, Double-Blind, Placebo-Controlled Study. CNS Drugs. 2018 Jul;32(7):661–672.	Biotin	1	-1
Gariballa S, Forster S. Effects of dietary supplements on depressive symptoms in older patients: a randomised double-blind placebo-controlled trial. Clin Nutr. 2007 Oct;26(5):545–51.	Biotin	5	+1
Muss C, Mosgoeller W, Endler T. Bioavailabilty of a liquid Vitamin Trace Element Composition in healthy volunteers. Neuro Endocrinol Lett.2015;36(4):337–47.	Biotin	5	+1
Sarris J et al. Participant experiences from chronic administration of a multivitamin versus placebo on subjective health and wellbeing: a double-blind qualitative analysis of a randomised controlled trial. Nutr J. 2012 Dec 14;11:110.	Biotin	5	+1
Harris E et al. Effects of a multivitamin, mineral and herbal supplement on cognition and blood biomarkers in older men: a randomised, placebo-controlled trial. Hum Psychopharmacol. 2012 Jul;27(4):370–7.	Biotin	5	0
Chew EY et al. Effect of Omega-3 Fatty Acids, Lutein/Zeaxanthin, or Other Nutrient Supplementation on Cognitive Function: The AREDS2 Randomized Clinical Trial. JAMA. 2015 Aug 25;314(8):791–801.	Carotenoids	3	-1
Yaffe K, et al. Impact of antioxidants, zinc, and copper on cognition in the elderly: a randomized, controlled trial. Neurology. 2004 Nov 9;63(9):1705–7	Carotenoids	2	-1
Gil Gregorio P et al. Dementia and Nutrition. Intervention study in institutionalized patients with Alzheimer disease. J Nutr Health Aging. 2003;7(5):304–8.	Carotenoids	5	+1
Sarris J et al. Participant experiences from chronic administration of a multivitamin versus placebo on subjective health and wellbeing: a double-blind qualitative analysis of a randomised controlled trial. Nutr J. 2012 Dec 14;11:110. doi: 10.1186/1475-2891-11-110.	Carotenoids	5	+1
Renzi-Hammond LM et al. Effects of a Lutein and Zeaxanthin Intervention on Cognitive Function: A Randomized, Double-Masked, Placebo-Controlled Trial of Younger Healthy Adults. Nutrients. 2017 Nov 14;9(11).	Carotenoids	1	+1
Lindbergh CA et al. Lutein and Zeaxanthin Influence Brain Function in Older Adults: A Randomized Controlled Trial. J Int Neuropsychol Soc. 2018 Jan;24(1):77–90.	Carotenoids	1	+1
Power R et al. Supplemental Retinal Carotenoids Enhance Memory in Healthy Individuals with Low Levels of Macular Pigment in A Randomized, Double-Blind, Placebo-Controlled Clinical Trial. J Alzheimers Dis. 2018;61(3):947–961	Carotenoids	1	+1
Hammond BR Jr et al. Effects of Lutein/Zeaxanthin Supplementation on the Cognitive Function of Community Dwelling Older Adults: A Randomized, Double-Masked, Placebo-Controlled Trial. Front Aging Neurosci. 2017 Aug 3;9:254.	Carotenoids	1	+1
Grodstein F et al. A randomized trial of β-carotene supplementation and cognitive function in men: the Physicians’ Health Study II. Arch Intern Med. 2007 Nov 12;167(20):2184–90.	Carotenoids	1	+1
McNeill G et al. Effect of multivitamin and multimineral supplementation on cognitive function in men and women aged 65 years and over: a randomised controlled trial. Nutr J. 2007 May 2;6:10.	Carotenoids	5	0
Stange I et al. Effects of a low-volume, nutrient- and energy-dense oral nutritional supplement on nutritional and functional status: a randomized, controlled trial in nursing home residents. J Am Med Dir Assoc. 2013 Aug;14(8):628.e1–8.	Carotenoids	5	0
Krikorian R et al. Improved cognitive-cerebral function in older adults with chromium supplementation. Nutr Neurosci. 2010 Jun;13(3):116–22.	Cr+3	1	+1
Davidson JR et al. Effectiveness of chromium in atypical depression: a placebo-controlled trial. Biol Psychiatry. 2003 Feb 1;53(3):261–4.	Cr+3	1	+1
Blake S. Hawaii Dementia Prevention Trial: A Randomized Trial Evaluating a Multifaceted Nutritional Intervention to Slow Cognitive Decline in Mild Cognitive Impairment Patients. Journal of Brain Sciences, Conscientia Beam 2018, 2(1), 1–12.	Cr+3	5	+1
Sarris J et al. Participant experiences from chronic administration of a multivitamin versus placebo on subjective health and wellbeing: a double-blind qualitative analysis of a randomised controlled trial. Nutr J. 2012 Dec 14;11:110.	Cr+3	5	+1
Henderson VW et al. Cognitive effects of estradiol after menopause: A randomized trial of the timing hypothesis. Neurology. 2016 Aug 16;87(7):699–708.	Oestrogen (E)	1	-1
Sherwin BB et al. A randomized controlled trial of estrogen treatment in men with mild cognitive impairment. Neurobiol Aging. 2011 Oct;32(10):1808–17.	Oestrogen (E)	1	0
Polo-Kantola P et al. The effect of short-term estrogen replacement therapy on cognition: a randomized, double-blind, cross-over trial in postmenopausal women. Obstet Gynecol. 1998 Mar;91(3):459–66.	Oestrogen (E)	1	-1
Henderson VW et al. Estrogen for Alzheimer’s disease in women: randomized, double-blind, placebo-controlled trial. Neurology. 2000 Jan 25;54(2):295–301	Oestrogen (E)	1	-1
Parkinson Study Group POETRY Investigators. A randomized pilot trial of estrogen replacement therapy in post-menopausal women with Parkinson’s disease. Parkinsonism Relat Disord. 2011 Dec;17(10):757–60.	Oestrogen (E)	1	0
Voskuhl RR et al. Estriol combined with glatiramer acetate for women with relapsing-remitting multiple sclerosis: a randomised, placebo-controlled, phase 2 trial. Lancet Neurol. 2016 Jan;15(1):35–46.	Oestrogen (E)	2	+1
Kocoska-Maras L et al. A randomized trial of the effect of testosterone and estrogen on verbal fluency, verbal memory, and spatial ability in healthy postmenopausal women. Fertil Steril. 2011 Jan;95(1):152–7.	Oestrogen (E)	1	-1
Tierney MC et al. A randomized double-blind trial of the effects of hormone therapy on delayed verbal recall in older women. Psychoneuroendocrinology. 2009 Aug;34(7):1065–74.	Oestrogen (E)	1	0
Bagger YZ et al. Early postmenopausal hormone therapy may prevent cognitive impairment later in life. Menopause. 2005 Jan-Feb;12(1):12–7.	Oestrogen (E)	1	+1
Mulnard RA et al. Estrogen replacement therapy for treatment of mild to moderate Alzheimer disease: a randomized controlled trial. Alzheimer’s Disease Cooperative Study. JAMA. 2000 Feb 23;283(8):1007–15.	Oestrogen (E)	1	-1
Yoon BK et al. Menopausal hormone therapy and mild cognitive impairment: a randomized, placebo-controlled trial. Menopause. 2018 Aug;25(8):870–876.	Oestrogen (P)	1	+1
De Giglio L et al. Effect on Cognition of Estroprogestins Combined with Interferon-β in Multiple Sclerosis: Analysis of Secondary Outcomes from a Randomised Controlled Trial. CNS Drugs. 2017 Feb;31(2):161–168.	Oestrogen (P)	2	+1
Moradi F et al. The effect of hormone replacement therapy on cognitive function in postmenopausal women: An RCT. Int J Reprod Biomed (Yazd). 2019 Jan 28;16(12).	Oestrogen (P)	1	+1
Gordon JL et al. Efficacy of Transdermal Estradiol and Micronized Progesterone in the Prevention of Depressive Symptoms in the Menopause Transition: A Randomized Clinical Trial. JAMA Psychiatry. 2018 Feb 1;75(2):149–157.	Oestrogen (P)	2	+1
Rapp SR et al. Effect of estrogen plus progestin on global cognitive function in postmenopausal women: the Women’s Health Initiative Memory Study: a randomized controlled trial. JAMA. 2003 May 28;289(20):2663–72	Oestrogen (P)	2	-1
Maki PM et al. Hormone therapy in menopausal women with cognitive complaints: a randomized, double-blind trial.Neurology. 2007 Sep 25;69(13):1322–30	Oestrogen (P)	1	-1
Gleason CE et al. Effects of Hormone Therapy on Cognition and Mood in Recently Postmenopausal Women: Findings from the Randomized, Controlled KEEPS-Cognitive and Affective Study. PLoS Med. 2015 Jun 2;12(6):e1001833; discussion e1001833	Oestrogen (P)	1	0
Almeida OP et al.. A 20-week randomized controlled trial of estradiol replacement therapy for women aged 70 years and older: effect on mood, cognition and quality of life. Neurobiol Aging. 2006 Jan;27(1):141–9.	Oestrogen (P)	1	-1
Pan HA et al. Cognitive function variations in postmenopausal women treated with continuous, combined HRT or tibolone. A comparison. J Reprod Med. 2003 May;48(5):375–80.	Oestrogen (P)	2	+1
Morrison MF et al. Lack of efficacy of estradiol for depression in postmenopausal women: a randomized, controlled trial. Biol Psychiatry. 2004 Feb 15;55(4):406–12	Oestrogen (P)	1	-1
Binder EF et al. Effects of hormone replacement therapy on cognitive performance in elderly women. Maturitas. 2001 Apr 20;38(2):137–46	Oestrogen (P)	2	-1
Baskaran C et al. Estrogen Replacement Improves Verbal Memory and Executive Control in Oligomenorrheic/Amenorrheic Athletes in a Randomized Controlled Trial. J Clin Psychiatry. 2017 May;78(5):e490-e497.	Oestrogen (T)	1	+1
Asthana S et al. Cognitive and neuroendocrine response to transdermal estrogen in postmenopausal women with Alzheimer’s disease: results of a placebo-controlled, double-blind, pilot study. Psychoneuroendocrinology. 1999 Aug;24(6):657–77	Oestrogen (T)	1	+1
Asthana S et al. High-dose estradiol improves cognition for women with AD: results of a randomized study. Neurology. 2001 Aug 28;57(4):605–12	Oestrogen (T)	1	+1
Wharton W et al. Short-term hormone therapy with transdermal estradiol improves cognition for postmenopausal women with Alzheimer’s disease: results of a randomized controlled trial. J Alzheimers Dis. 2011;26(3):495–505.	Oestrogen (T)	1	+1
Kulkarni J et al. Estradiol for treatment-resistant schizophrenia: a large-scale randomized-controlled trial in women of child-bearing age. Mol Psychiatry. 2015 Jun;20(6):695–702	Oestrogen (T)	1	+1
Schiff R et al. Short-term transdermal estradiol therapy, cognition and depressive symptoms in healthy older women. A randomised placebo controlled pilot cross-over study. Psychoneuroendocrinology. 2005May;30(4):309–15	Oestrogen (T)	1	0
Joffe H et al. Estrogen therapy selectively enhances prefrontal cognitive processes: a randomized, double-blind, placebo-controlled study with functional magnetic resonance imaging in perimenopausal and recently postmenopausal women. Menopause. 2006 May-Jun;13(3):411–22.	Oestrogen (T)	1	+1
Kryscio RJ et al. Association of Antioxidant Supplement Use and Dementia in the Prevention of Alzheimer’s Disease by Vitamin E and Selenium Trial (PREADViSE). JAMA Neurol. 2017 May 1;74(5):567–573.	Se	2	-1
Corrigan F et al. Dietary Supplementation with Zinc Sulphate, Sodium Selenite and Fatty Acids in Early Dementia of Alzheimer’s Type. II: Effects on Lipids. Journal of Nutritional & Environmental Medicine. 1990; 2. 265–271.	Se	3	+1
Scheltens P et al. Efficacy of Souvenaid in mild Alzheimer’s disease: results from a randomized, controlled trial. J Alzheimers Dis. 2012;31(1):225–36.	Se	5	+1
Blake S. Hawaii Dementia Prevention Trial: A Randomized Trial Evaluating a Multifaceted Nutritional Intervention to Slow Cognitive Decline in Mild Cognitive Impairment Patients. Journal of Brain Sciences, Conscientia Beam 2018, 2(1), 1–12.	Se	5	+1
Muss C, Mosgoeller W, Endler T. Bioavailabilty of a liquid Vitamin Trace Element Composition in healthy volunteers. Neuro Endocrinol Lett.2015;36(4):337–47	Se	5	+1
Cornelli U. Treatment of Alzheimer’s disease with a cholinesterase inhibitor combined with antioxidants. Neurodegener Dis. 2010;7(1–3):193–202.	Se	5	+1
Sarris J et al. Participant experiences from chronic administration of a multivitamin versus placebo on subjective health and wellbeing: a double-blind qualitative analysis of a randomised controlled trial. Nutr J. 2012 Dec 14;11:110.	Se	5	+1
Kesse-Guyot E et al. French adults’ cognitive performance after daily supplementation with antioxidant vitamins and minerals at nutritional doses: a post hoc analysis of the Supplementation in Vitamins and Mineral Antioxidants (SU.VI.MAX) trial. Am J Clin Nutr. 2011 Sep;94(3):892–9.	Zn+2	5	+1
Blake S. Hawaii Dementia Prevention Trial: A Randomized Trial Evaluating a Multifaceted Nutritional Intervention to Slow Cognitive Decline in Mild Cognitive Impairment Patients. Journal of Brain Sciences, Conscientia Beam 2018, 2(1), 1–12.	Zn+2	5	+1
Yaffe K, et al.. Impact of antioxidants, zinc, and copper on cognition in the elderly: a randomized, controlled trial. Neurology. 2004 Nov 9;63(9):1705–7	Zn+2	2	-1
Brewer GJ. Alzheimer’s disease causation by copper toxicity and treatment with zinc. Front Aging Neurosci. 2014 May 16;6:92.	Zn+2	1	+1
Scheltens P et al. Efficacy of a medical food in mild Alzheimer’s disease: A randomized, controlled trial. Alzheimers Dement. 2010 Jan;6(1):1–10.e1.	Zn+2	5	+1
Stewart-Knox BJ et al. Supplemented zinc does not alter mood in healthy older European adults—a randomised placebo-controlled trial: the Zenith study. Public Health Nutr. 2011 May;14(5):882–8.	Zn+2	1	-1
Gil Gregorio P et al. Dementia and Nutrition. Intervention study in institutionalized patients with Alzheimer disease. J Nutr Health Aging. 2003;7(5):304–8.	Zn+2	5	+1
Gariballa S, Forster S. Effects of dietary supplements on depressive symptoms in older patients: a randomised double-blind placebo-controlled trial. Clin Nutr. 2007 Oct;26(5):545–51.	Zn+2	5	+1
Veasey RC et al. The Effects of Supplementation with a Vitamin and Mineral Complex with Guaraná Prior to Fasted Exercise on Affect, Exertion, Cognitive Performance, and Substrate Metabolism: A Randomized Controlled Trial. Nutrients. 2015 Jul 27;7(8):6109–27.	Zn+2	5	+1
Muss C, Mosgoeller W, Endler T. Bioavailabilty of a liquid Vitamin Trace Element Composition in healthy volunteers. Neuro Endocrinol Lett.2015;36(4):337–47	Zn+2	5	+1
Sarris J et al. Participant experiences from chronic administration of a multivitamin versus placebo on subjective health and wellbeing: a double-blind qualitative analysis of a randomised controlled trial. Nutr J. 2012 Dec 14;11:110	Zn+2	5	+1
McNeill G et al. Effect of multivitamin and multimineral supplementation on cognitive function in men and women aged 65 years and over: a randomised controlled trial. Nutr J. 2007 May 2;6:10.	Zn+2	5	0

The columns indicate source, the active agent, modality of the trial (MOD) denoting how many components were in a cocktail, or if that was a single substance trial, and self-assessment incremental success metric: +1 –positive, 0 –neutral, -1 –negative.

**Table 3:** Results of multiple regression analysis linking the potential contributing factors (A, C) with cognitive status, as well as linking the potential confounders and the presence of multiple protective exposures (B, D) in personal profiles. Only significant factors with p < 0.1 are reported, with p-values generated by LINEST software.

**A. Low MOCA < 20 as an outcome in NSHAP, contributing factors, (+) putatively facilitate cognitive decline; (-) putatively oppose cognitive decline**.						
	*Coefficients*	*Standard Error*	*t Stat*	*P-value*	*Lower 95%*	*Upper 95%*
Intercept	0.199	0.125	1.597	0.110	-0.045	0.444
MULTIPLE PROTECTANTS	-0.021	0.004	-5.199	0.000	-0.029	-0.013
EDUCATION	-0.073	0.008	-9.726	0.000	-0.088	-0.058
GOOD VISION AND HEARING	-0.019	0.005	-3.751	0.000	-0.029	-0.009
GOOD PHYSICAL HEALTH, SELF-ASSESSED	-0.022	0.011	-2.083	0.037	-0.043	-0.001
GOOD MENTAL HEALTH; SELF-ASSESSED	-0.020	0.011	-1.909	0.056	-0.041	0.001
ALCOHOL SOCIALLY CONSUMED	-0.086	0.019	-4.551	0.000	-0.124	-0.049
OSTEOARTHRITIS	-0.076	0.023	-3.232	0.001	-0.122	-0.030
HAPPY, CONFIDENT, RELAXED	-0.006	0.002	-3.339	0.001	-0.009	-0.002
COGNITIVE ERRORS AT BASELINE	0.168	0.016	10.741	0.000	0.137	0.198
AGE	0.012	0.001	9.797	0.000	0.010	0.015
**B. Multiple supplements as an outcome in NSHAP, potential factors either favoring (+) or opposing (-) the presence of protectants in personal profiles**						
	*Coefficients*	*Standard Error*	*t Stat*	*P-value*	*Lower 95%*	*Upper 95%*
Intercept	-2.124	0.670	-3.169	0.002	-3.439	-0.809
EDUCATION	0.156	0.040	3.861	0.000	0.077	0.235
PRESCRIPTION MEDICINES	0.072	0.015	4.760	0.000	0.042	0.101
GOOD PHYSICAL HEALTH; SELF ASSESSED	0.105	0.057	1.830	0.067	-0.007	0.217
GOOD MENTAL HEALTH; SELF ASSESSED	0.148	0.057	2.606	0.009	0.037	0.259
HOURS IN BED	-0.054	0.021	-2.621	0.009	-0.095	-0.014
CURIOUS	0.143	0.058	2.465	0.014	0.029	0.256
PHYSICALLY ACTIVE	0.061	0.027	2.220	0.027	0.007	0.115
ALCOHOL SOCIALLY CONSUMED	0.248	0.102	2.422	0.016	0.047	0.448
OSTEOARTHRITIS	0.471	0.126	3.747	0.000	0.225	0.718
AGE	0.027	0.007	3.984	0.000	0.014	0.040
**C. Dementia as an outcome in NACC, contributing factors, (+) putatively facilitate cognitive decline; (-) putatively oppose cognitive decline**.						
	*Coefficients*	*Standard Error*	*t Stat*	*P-value*	*Lower 95%*	*Upper 95%*
Intercept	1.342	0.020	68.427	0.000	1.304	1.381
POLYPHARMACY AT THE END OF FOLLOW UP	0.012	0.001	20.577	0.000	0.011	0.013
GENDER (1—F, 0-M)	-0.057	0.004	-13.289	0.000	-0.065	-0.048
EDUCATION, YEARS	-0.003	0.001	-4.577	0.000	-0.004	-0.002
OSTEOARTHRITIS AT THE END OF FOLLOW UP	-0.061	0.003	-18.144	0.000	-0.067	-0.054
AGE AT BASELINE	0.004	0.000	18.581	0.000	0.003	0.004
DEMENTIA AT BASELINE	-0.045	0.000	-122.227	0.000	-0.045	-0.044
FOLLOW-UP, YEARS	0.017	0.001	23.073	0.000	0.016	0.019
MULTIPLE PROTECTANTS	-0.016	0.001	-17.700	0.000	-0.017	-0.014
**D. Multiple supplements as an outcome in NACC, potential factors either favoring (+) or opposing (-) the presence of protectants in personal profiles**						
	*Coefficients*	*Standard Error*	*t Stat*	*P-value*	*Lower 95%*	*Upper 95%*
Intercept	-1.956	0.112	-17.465	0.000	-2.175	-1.736
POLYPHARMACY AT THE END OF FOLLOW UP	0.317	0.003	112.782	0.000	0.311	0.322
GENDER (1—F, 0-M)	0.606	0.024	25.009	0.000	0.559	0.654
EDUCATION, YEARS	0.081	0.004	22.955	0.000	0.074	0.088
AGE AT BASELINE	-0.015	0.001	-13.393	0.000	-0.018	-0.013
MMSE COGNITIVE SCORE AT BASELINE	0.041	0.002	19.481	0.000	0.037	0.045
FOLLOW-UP, YEARS	0.237	0.004	57.329	0.000	0.229	0.245

A. The results of regression in NSHAP data, 2138 respondents, the outcome is the presence of MOCA score < 20. The factors are defined in the NSHAP codebook attached to the report in Supplemental materials. “Coefficients” indicate the weights in linear regression (LINEST), with the residual minimized by least square method. T-Stat is defined by LINEST function of EXCEL as the ratio of the coefficient and standard error. P-value of the T-stat are provided, as well as confidence intervals CI95 for the coefficients.

B. The results of regression in NSHAP data, 2138 respondents, the outcome is the number of protectants in personal profiles, the coefficients are determined for the contribution of factors that either parallel or counter the presence the multiple protectants in personal profiles.

C. The analysis analogous to A and conducted in NACC, 38838 patients, the outcome is fraction of dementia at the end of follow up.

D. The analysis analogous to B and conducted in NACC, 38838 patients, the outcome is the number of protectants in personal profiles.

**Table 4:** Dependence of the cognitive and survival outcomes on the number of protective compounds reported in the patient’s personal profile.

DB	N	% rank	PAT	FP (Y)	DEM B	DEM E	STR	Age B	Age E	LS	AMRT
NACC1	4.5	0–2%	120	5.2	**0.08**	**0.19**	0.058	72.3	78.1	86.8	0.14
NACC1	3.5	0–5%	300	4.9	**0.11**	**0.23**	0.061	72.25	77.1	87.3	0.16
NACC1	2	5–26%	1300	5.1	**0.18**	**0.33**	0.08	72.61	77.8	86.6	0.19
NACC1	1	27–51%	1500	5.2	**0.23**	**0.41**	0.085	72.47	78.7	85	0.22
NACC1	0.2	52–100%	3000	5.1	**0.34**	**0.52**	0.087	72.16	78.3	84.3	0.29
NACC2	2.6	0–12%	400	2.2	**0.32**	**0.44**	0.11	88.03	90.3	93.7	0.39
NACC2	0.4	13–100%	3400	2.2	**0.44**	**0.59**	0.14	88.34	90.7	93.5	0.49
NSHAP	2.5	0–14%	300	5	**0.27**	**0.31**	0.072	67.7	72.8	72.5	0.10
NSHAP	1.2	15–52%	800	5	**0.35**	**0.44**	0.097	68.8	74	72.8	0.13
NSHAP	0	53–100%	1040	5	**0.42**	**0.64**	0.092	68.2	73.2	72.4	0.16
NAMCS	2.8	0–6%	300	NA	**0.018**	**NA**	0.05	73.2	NA	NA	NA
NAMCS	1.5	7–31%	1500	NA	**0.029**	**NA**	0.058	73.2	NA	NA	NA
NAMCS	0.5	32–70%	1700	NA	**0.039**	**NA**	0.069	72.9	NA	NA	NA
NAMCS	0	71–100%	1500	NA	**0.051**	**NA**	0.072	72.9	NA	NA	NA
**Confounding factors in NACC population**.											
**Parameter**					**Highly protected cohort**				**Control**		
Fraction of females					0.58				0.55		
Fraction of advanced degree holders					0.35				0.32		
Comorbidity at the beginning of follow-up (FP)					2.3				2.6		
Comorbidity at the end of follow-up					5.9				5.8		
Cardiovascular at the beginning					1.0				1.1		
Cardiovascular at the end					2.0				1.9		
Metabolic syndrome at the beginning					1.0				1.0		
Metabolic syndrome at the end					3.4				3.2		
Diabetes at the end					0.19				0.195		

Due to different degrees of detected confounding effects, analysis of NACC datasets excluded arthritis patients, while in NSHAP and NAMCS datasets this confounder was controlled.

NACC1 –cohort with males excluded, arthritis excluded, follow-up 5 years, 6100 patients; NACC2 –cohort with ages 88–90, 3740 patients; NSHAP–all categories included, 2140 patients linked by ID and present in both the 2005–2006 and 2010–2011 waves of the survey, 5 year follow-up; NAMCS–all categories included, ages above 60 years.

DB–database source, N–number of protectants in the personal profile, % rank–the percent of the total rank, beginning at the top; the lower the number is, the closer the stratum is to the top of the ranking, PAT–the number of patients in the cohort, FP–follow-up length in years, DEM B–dementia fraction at the beginning of follow-up, DEM E–dementia fraction at the end of follow-up, STR–stroke at the end of follow-up, Age B–age at the beginning of follow-up, Age E–age at the end of follow-up, LS–recorded lifespan, AMRT–all cause mortality, fraction.

DEM B and DEM E in the NSHAP data are represented by the numbers of errors in cognitive tests corresponding to the pre-MCI and MCI stages of cognitive decline, respectively. AMRT is measured during follow-up between AGE B and AGE E, and STR is measured at AGE E.

**Table 5:** Statistical analysis of outcomes (O:) and confounders (C:) as a function of exposure to different levels of protectant diversity in the NACC release as of 09/2018, version 3.

	General population			Decedent component		
Percentile from the top of the rank by protectants	0–5%	51–100%	p-value	0–5%	51–100%	p-value
NG	800	7600	-	-	-	-
ND	-	-	-	52	643	-
PR	5.35	1.02	0	5.25	1.05	1.6x10^-131
O:FP	6.5	3.5	6.8x10^-105	6.48	5.15	0.011
O:FPB	69.3	72.5	4.2x10^-23	75.2	77.2	0.15
C:FPE	75.8	76	0.45	81.6	82.4	0.67
**O:DEM1**	**0.08**	**0.24**	**5.9x10^-25**	**0.25**	**0.55**	**3.3x10^-5**
**O:DEM2**	**0.2**	**0.33**	**1.75x10^-14**	**0.56**	**0.76**	**9.7x10^-4**
**O:MMSE1**	**29.11**	**27.48**	**9.3x10^-23**	**27**	**24.5**	**0.006**
**O:MMSE2**	**26.8**	**24.8**	**1.3x10^-18**	**22.5**	**19.6**	**0.01**
O:AMRT	0.065	0.084	0.063	-	-	-
O:LS	82.6	80	0.24	81.8	82.4	0.68
O:LE	7.5	3.6	0.06	-	-	-
O:CANC	0.24	0.21	0.044	0.39	0.26	0.081
O: ΔMMSE/FP	-0.31	-0.36	0.07	-0.7	-0.8	0.53
O: ΔDEM/FP	+0.025	+0.032	0.075	+0.046	+0.045	0.93
**O: H(T)**	**0.16**	**0.267**	**0.007**	**-**	-	-
C: FEM	0.63	0.61	0.26	0.42	0.51	0.21
C: EDUC	3.18	3.0	0.49	3.01	3.06	0.96
C: ARTH	0.65	0.63	0.39	0.49	0.59	0.16
C: DIAB	0.14	0.14	0.91	0.19	0.13	0.26
C:CARD	0.58	0.59	0.86	0.65	0.52	0.26
C:TOT	2.58	2.56	0.75	4.07	3.12	0.008
C: POLY	4.75	4.66	0.39	6.04	5.2	0.038

The rows 1 and 2 refer to the numbers of patients in the corresponding cohorts, NG–number in the general population, ND–number of decedents. The row 3 refers to the number of protectants per a patient’s profile in the cohort.

Outcomes: FP–follow up, years, FPB–age of entering observation, years, FPE- age of ending observation, years, DEM1, DEM2 –fractions of dementia at the beginning and end of follow up respectively, MMSE1, MMSE2 –average MMSE in the cohort at the beginning and at the end of follow up respectively, AMRT–all-cause mortality fraction accrued during observation started at 100% survival in the elderly database population, LS–lifespan in the group, years, LE–life expectancy, years between start of observation and end of life, D MMSE/FP–time derivative of MMSE per a person, units of score per year, D DEM/FP–time derivative of dementia fraction per a person, fraction change per year, H(T)–hazard function for mortality next year, estimated as AMRT/(LS–FBP).

Confounders: FEM–percent of females in the group, EDUC–years of advanced degree education per a member of the group, ARTH–fraction of osteoarthritis in the group at the end of follow up, DIAB–fraction of diabetes in the group at the end of follow up, CARD–fraction of serious cardiovascular disease at the end of follow up, the list includes infarction, congestive heart failure, angioplasty, stents, fibrillation events, stroke, angina, TOT–total multi-morbidity at the end of follow up, POLY–non-protective polypharmacy at the end of follow up.

The columns 2–4 refer to the general population (G) and the columns 5–7 refer to the decedents (D) formed in the respective populations. The same outcomes and confounders were re-measured in both general and decedent populations, but confounder normalization was conducted only in general population, leaving the same metrics to float and accept the final value in the decedents. The columns 1–5% refer to the top 1–5% of rank by the protectants, 51–100% refer to the bottom 51–100% rank by the protectants.

The columns from 2 to 7:

2. 0–5%—outcome and confounder values in the cohort formed by the top 0–5% of the rank in the general population by the number of protectants in the patient’s profile.

3. 51–100%—the same as column 2, but values in the bottom rank 51–100%.

4. p-values assessing significance of differences between the 0–5% and 51–100% cohorts.

5–7 –the same as columns 2–4 (which are measured in living population) but measured for the decedents formed in the general population.

The agents tested as protectants included any of: oestrogens, zinc, melatonin, creatine, choline, serine, arginine, lysine, selenium, calcium-vitamin D, chromium picolinate, biotin, herbal supplements, vitamin A and lutein supplement, chondroitin and glucosamine, magnesium, omega-3.

The most important outcomes are marked in bold (H(T), DEM, MMSE).

**Table 6:** Statistical analysis of outcomes (O:) and confounders (C:) as a function of exposure to different levels of protectant diversity in the NACC release as of 09/2018, version 3.

	General population			Decedent component		
Percentile from the top of the rank by protectants	0–2%	3–100%	p-value	0–2%	3–100%	p-value
NG	300	15300	-	-	-	-
ND	-	-	-	14	1236	-
PR	2.99	0.38	0	3.2	0.36	5.3x10^-49
O:FP	5.66	4.2	4.9 x 10^-11	7.2	5.4	0.0018
O:FPB	69.75	70.62	0.22	74.3	75.21	0.44
O:FPE	75.4	74.8	0.19	81.5	80.3	0.086
**O:DEM1**	**0.04**	**0.14**	**2.4 x 10^-10**	**0.06**	**0.51**	**1.7x10^-5**
**O:DEM2**	**0.21**	**0.31**	**2.1 x 10^-7**	**0.26**	**0.76**	**1.1 x10^-8**
**O:MMSE1**	**29.23**	**28.0**	**8.8 x10^-6**	**27.7**	**25.2**	**0.013**
**O:MMSE2**	**27.14**	**25.4**	**1.8 x 10^-5**	**22.8**	**19.9**	**0.021**
O:AMRT	0.05	0.085	0.016	-	-	-
O:LS	-	-	-	82.5	81.4	0.21
O:LE				8.26	6.26	0.009
O:CANC	0.199	0.212	0.42	0.46	0.27	0.21
O: ΔMMSE/FP	0.28	0.35	0.18	0.7	0.85	0.23
O: ΔDEM/FP	0.017/y	0.024/y	-	0.078/y	0.112/y	-
**O: H(T)**	**0.0088/y**	**0.020/y**	**-**	**-**	**-**	**-**
C: FEM	0.64	0.58	0.83	0.46	0.47	0.26
C: EDUC	1.02	0.95	0.86	1.26	0.86	0.88
C: ARTH	0.63	0.57	0.077	0.66	0.50	0.38
C: DIAB	0.32	0.29	0.44	0.26	0.27	0.46
C:CARD	0.56	0.58	0.82	1.26	1.06	0.89
C:TOT	2.49	2.53	0.71	3.7	3.1	0.66
C: POLY	4.85	4.46	0.080	5.25	5.29	0.89
**Comparison of exposures with equal complexity but different average REG and correlation of these exposures with dementia rates for 9203 decedents identified in NACC**.						
**Metric**		**Composition 1**	**Composition 2**	**Control**	**p-value 1/2**	**p-value 1/control**
ND (all are decedents, 9203)		40	320	8443	-	-
C:Total polypharmacy, agents		14.13	13.03	7.11	0.0211	1.01x10^-28
C: Prescription polypharmacy, agents		4.7	4.9	5.4	0.58	0.14
C: OTC supplement agents		11.48	9.42	2.58	4.75x10^-23	7.44x10^-139
O:FPB		79.24	76.4	76.0	0.055	0.040
O:FPE		85.12	82.17	78.7	0.045	0.0002
**O:DEM1**		**0.0975**	**0.32**	**0.63**	**0.00096**	**1.9x10^-12**
**O:DEM2**		**0.317**	**0.658**	**0.767**	**1.86x10^-5**	**1 x 10^-11**
**O:MMSE1**		**28.17**	**26.78**	**22.21**	**0.0045**	**1.06 x10^-6**
**O:MMSE2**		**23.05**	**20.46**	**17.93**	**0.03**	**0.00018**
O:LS		86.9	83.9	80.74	0.04	0.00039
O:LE		7.65	7.51	4.73	0.58	1.65x10^-10
O:CANC		0.307	0.293	0.267	0.769	0.659
C: FEM		0.63	0.51	0.46	0.195	0.034
C: EDUC		14.65	15.83	14.8	0.021	0.78
C: ARTH		0.268	0.185	0.056	0.33	1.5x10^-8
C: DIAB		0.146	0.135	0.123	0.81	0.65
C: CARD		1.609	1.345	0.99	0.304	0.022

See the definitions of Table 5 for details of notations. The difference between the Tables 5 and 6 data is the composition. The agents tested as protectants included any of: zinc, selenium, chromium picolinate, biotin, herbal supplements, vitamin A and lutein supplement, angiotensin-receptor blockers.

For the addendum part, 9203 decedents only were included in the analysis, produced in all NACC versions. Composition 1 (high complexity, higher REG agents as per Table 1) includes any of: angiotensin receptor blockers, NSAIDs, anti-diabetics, selenium, biotin, chromium picolinate, zinc, Vitamin A + lutein, garlic, red yeast rice, turmeric, cranberry, flax, the rest are any of the prescription medications. Composition 2 (high complexity, lower REG agents as per Table 1) includes any of: ubiquinone, multivitamins, omega-3, vitamin D, folic acid, vitamin B12, vitamin E, vitamin C, calcium dosage forms, probiotics, the rest are any of the prescription medications. Control are the remaining profiles.

Cohort with prevailing composition 1 occupies profiles 1–40 on the top of the rank, the composition 2 occupies the positions 41–320 from the top and the control occupies the positions 321–9208 of the rank. The boundaries of the categories were selected to maintain the number of OTC components ~ 10 for both compositions, maximal for this database enabling to assess the upper limit of the effects.

The most important outcomes are marked in bold (H(T), DEM, MMSE).

The National Social Life, Health, and Aging Project (NSHAP) Waves 1 and 2 [18] are affiliated with the Inter-university Consortium for Political and Social Research. The longitudinal dataset (>3000 patients) provides information about supplement and pharmaceutical exposure, comorbidity presence and all-cause mortality. The dementia rate is available but is under-represented compared to the age-matched general population outside of the survey. Cognitive scores were used instead.

The National Ambulatory Medical Care Surveys (NAMCS) in 2009 collected data on ICD-9 medical codes, including dementia and medications, for more than 32,000 ambulatory visits to 1,283 doctor’s offices [19]. This study utilized 11,000 profiles of the elderly, after discarding the profiles with ages < 60.

### Supplemental data

The original and processed datasets have been uploaded and tabulated. The summary describing the published datasets with the links is provided in the Table of Supplemental Files. Individual supplemental files are given in the text as links, illustrating the selected points. The File A in S1 Data is accompanied with the demos explaining data flow.

### Forming groups based on individual active compounds

All databases mentioned in the Data Sources are relational, with the rows linked to the patient ID and the columns designated for comorbidities, treatments, age, gender, race and lifestyle parameters. In case of NACC, multiple rows may represent dated visits by the same patient (38000 patients). All components in the datasets can be clustered using Pivot Tables function of Microsoft Excel, aggregating numerical and non-numerical identifiers linked to the given identifier in the same row. The groups analysed in Table 1 of the Results were formed by Pivot Table clustering of names for the dosage forms (FDA approved pharmaceuticals and OTC supplements) available in NACC in > 134000 visit profiles. All other metrics associated with the patients receiving a given dosage form can be also summarized using the same tool: the counts of dementia tags, the count of MCI tags, average MMSE scores, the counts of other actives and comorbidities. These counts can be related to the number of visits in the cluster, and the result can be related to the same measurement for the control, producing hazard ratios reported in Table 1 of Main Manuscript. Versatile nature of the Pivot Table tool allows labelling of dosage forms as members of the same pharmacological mechanism and further aggregation of individual dosage forms in pooled groups based on mechanistic commonality, using the label for the aggregate as the grouping element. General flow of the data analysis is presented at Fig 1.

### Computing database metrics for aligning with clinical trials

[HRDEM], [HRNORM], [HRAMRT]–hazard ratios respectively of dementia, cognitive norm and all-cause mortality in groups formed based on receiving a treatment or agent and normalized to the entire database control. When data were available, another a-priori parameter [MOD] (modality) was applied, defined as the number of agents/factors/mechanisms simultaneously tested in a clinical trial.

### Screening the groups formed by a single factor for non-random origin of the signal

The tags for dementia (DEM), cognitive norm (NORM) and all-cause mortality (AMRT) were assumed not to follow normal distribution when considering grouping by actives. Random grouping was imposed by producing random tags and aligning this profile of 134000 numbers in the 1–300 range with 134000 visit profiles in NACC. The same Pivot Table procedure applied for combining the profiles based on a common agent was applied to combining according to the same random number. In the groups with the same random number, the parameters for dementia, norm and mortality were computed and variation between the groups was computed as well. Variation was assessed individually for HRDEM, HRNORM and HRAMRT, and for the combination of these metrics in the parameter REG (below) for each random group. These randomly derived standard deviations were adjusted to the size of the groups and served to form Z-scores:

$$Z=\sqrt{N/460}\;(FG-FC)/S$$ (1)

Where Z–z-score, $\sqrt{N/460}$–adjustment for the size of the groups N different from 460 used in random model calibration, FG–frequency of the marker in the group measured in relative units of hazard ratios, FC–frequency of the marker in the total population control in relative units (FC = 1), S = σ x t–Studentized variance, σ standard deviation produced in the random model, t–correction coefficient in Student’s distribution, a function of allowed reliability for the test (α< 0.1) and degrees of freedom (1). The correction coefficient is 6.314 in these assumptions for a two-sided test. The studentized Z-score > 1 in absolute values means > 90% probability of non-random origin of the effects irrespective to normal distribution of the effects. DEM, NORM, AMRT, REG can be the markers of interest.

### Meta-analysis of clinical trials and derivation of a classifier predicting clinical trial performance with a-priori inputs from the databases of electronic medical records

Clinical trials relating to prevention of neurodegeneration were selected for the training set. The trials included prevention of subjective or mild cognitive impairment (MCI), treatment of mild dementia, treatments of multiple sclerosis, Parkinson’s disease, resistant depression, psychoses. The trials directed to treatment of severe dementia were included as well but known anti-Alzheimer’s drugs and anti-psychotics were not considered due to lack of relevance to prevention. The focus of the effort was re-purposing of non-dementia directed FDA-approved agents and OTC supplements. The training set included > 250 elementary trials, presented in the (File C in S1 Data), collected for the period 2007–2017, using PubMed as a search engine.

The PubMed IDs of the trials were tabulated, the outcomes classified as success if the authors state the original hypothesis was confirmed (RCT+ = 1), failing if the authors state so (RCT- = 1) or otherwise neutral (RCT 0 = 1). Multiple components tested in the same trial were classified according to relevant general mechanisms (NSAID, antihistamine, hormone etc.) and the level of modality was noted (MOD = 1 –a single agent, MOD = 2–4–2–4 agents, MOD = 5 –five or above). These trials were attributed to 53 major groupings of pharmacological mechanisms defined either in a standard manner or according to the composition details provided in Results. The difference BAL between positive and negative results was normalized to total number of trials in a given mechanism (TOT). This metric (derived on clinical trial side) was correlated to REG (derived on EMR database side)–a linear combination of interpretable generic features, not depending on specifics of a mechanism, with the regression coefficients derived by optimizing Pearson correlation with the outcomes BAL/TOT.

$$\begin{array}{l} REG=1\left(\pm 0.13\right)x\left[HRNORM\right]-0.45\left(\pm 0.05\right)x\left[HRDEM\right]+0.2\left(\pm 0.021\right)x\left[AMRT\right]+\\ 0.15\left(\pm 0.018\right)x\left[MOD\right]-0.35\left(\pm 0.041\right)x\left[\frac{HRDEM}{AMRT}\right]+0.2\left(\pm 0.025\right)x\frac{\left[HRNORM\right]}{\left[AMRT\right]}+\\ 0.25\left(\pm 0.032\right)x\frac{\left[HRDEM\right]}{\left[HRNORM\right]}–0.02\left(\pm 0.023\right)x\frac{\left[MOD\right]}{\left[HRNORM\right]} \end{array}$$ (2)

Indeed, a strong dementia-preventing agent should increase hazard ratio of cognitive norm [HRNORM], decrease dementia hazard ratio [HRDEM], minimize mortality rate [AMRT], minimize the ratio of dementia to mortality rate [HRDEM]/[HRAMRT], be compatible with multimodal use [MOD], increase hazard ratio for normal cognitive status normalized to mortality [HRNORM]/[HRAMRT], be active at more advanced pathological process represented by a higher [HRDEM]/[HRNORM], require less multimodality to ensure a given hazard ratio of norm producing negative [MOD]/[HRNORM].

To test stability and generalization by the predictor, the training set was permuted by randomly excluding 10 elements on the BAL/TOT side and the resulting variation in the prediction rule allowed to compute variations in the regression coefficients in (2), providing 5-fold cross-validation. Generalization was assessed by variation in the resulting correlation coefficient between permuted BAL/TOT and adjusted REG profiles. Generalization was considered acceptable if the excluded elements retain the original rank being outside of the initial prediction rule. The performance of the predictor was assessed by Response Operative Characteristic (ROC), using the average permuted score as a ranking function and presenting BAL/TOT as either + 1 if BAL/TOT > 0 (positives) and -1 if BAL/TOT = 0 or < 0 (negative). Variation of the point’s locations due to permutation was reflected on the ROC plotting (Fig 2).

The predictor was assessed for signal-to-noise ratio by randomly scrambling BAL/TOT profiles (12 times) and the scrambled profile was re-correlated with REG.

The predictor was tested on a new set of 70 clinical trials identified by searching a different interface (Google Scholar) and representing clinical performance of the top octile by REG rank. The agents of interest were identified in the tables of compositions tested in the trials and the results were digitized identically to the training set. The BAL/TOT ratios for the agents were computed and compared with the profile of ratios for the training set. The ability to predict predominantly successful clinical trials and the ability to extrapolate continuous strong results based on initial promise were explored as validation tests.

### Forming groups based on simultaneous presence of multiple agents

With the ability of Regression scores (REG) to predict increased probability of trial success being confirmed, both REG and empirical trial performance were used to select a limited number of agents for combinational study. The candidates passing this double filter were traced in the individual profiles in NACC and counts PR (protectants) or N were produced. In some patients, PR was 0 (no protectants) and in some as high as 24 protective molecules consumed by a patient. Using Pivot Tables, visits in NACC were aggregated for each patient ID, producing the data point at the first and at the last visit during follow up. These data points were available for all parameters (cognitive, comorbidity, polypharmacy, protectants, duration of the follow up for each patient being a variable in NACC) tracked in this study for the first and last visit. In other datasets these metrics were pre-computed by the providers–such as the number of supplements per a profile in NSHAP. If validated agent on the short list was present in the profile during the final visit–PR was incremented by 1. The presence of the next agent on the short list of interest was producing the next increment etc.

### Normalization to confounding factors

Multiple regression was chosen as the major normalization method due to complex interactions between the confounding factors [20, 21]. The program LINEST is available as a supplement to Microsoft Excel [22]. Statistical significance of the regression coefficient for a factor X analysed in the context of all known confounders included in the model (p-value < 0.1) and its expected direction were the criteria for conclusion that the factor is independent. The data of Table 3, Figs 3 and 4 were normalized using multivariate regression.

With Bayesian competition hypothesis proposing reduction of dementia in multimorbid patients (Discussion) it was desirable to individually equalize all confounders between the case and control cohorts. For the given system:

1-HR(DEM)=W1x(PRcase-PRcont) (3)

Where 1 –HR(DEM) is deviation of the hazard ratio of dementia in a given case stratum of database with higher PR_case_ and the stratum with the lower PR_cont_ used as a control, where PR is the number of protectants per the profile, where W1 is non-adjusted regression coefficient. Considering the differences between the confounder levels in the case and control strata expands the regression:

1-HR(DEM)=W1x(PRcase-PRcont)+∑WiΔXi (4)

Where ∑WiΔXi is the contribution of the confounders Xi in the observed outcome, W’1 is confounder-adjusted value of the primary regression coefficient. A transformation of ranks can be introduced that nullifies ∑WiΔXi term and each ΔXi individually:

$$1-HR(DEM)=W1\;x\;(PRcase-PRcont+\;\Delta PR)=W^{\prime }\;1\;x({PR}^{\prime }case-{PR}^{\prime }cont)$$ (5)

Measuring the modified (PR’_case_ − PR’_cont_) and adjusted 1 –HR’(DEM) after the transformation allows to identify the confounder-adjusted regression coefficient W’1 linking the difference in the number of protectants and decrease in dementia in the case cohort. Combining (3)–(5) allows deconvolution of the roles of the main factor and the combination of confounders.

The rank transformation used in this report was the following:

$$PR^{\prime }=PR\;+\;\sum Vi\Delta Xi$$ (6)

Where is PR’–adjusted rank for a given patient profile according to the number of protectants, PR–initial non-adjusted rank, X_i_ − confounder values, V_i_ − variable weight coefficients. These weight coefficients were subjected to an optimization process with the criterion of ∑WiΔXi = 0; ΔXi, j, k = 0 as the convergence condition. The coefficients Vi were incrementally varied and after each variation the database was re-ranked and convergence criteria re-tested. Equalization of readings for all confounders accompanies the results reported in Tables 4–6.

### Choice of metrics to report the outcomes in the cohorts

With the cohorts of the fixed size, rare tag occurrence may produce random fluctuations exceeding the size of the intended measured effect. The tags were screened for the adequate prevalence in the cohort to report the effects with maximized signal-to-noise ratio.

$$\left[Cohort\;size]>NC=Z^{2}x(\frac{\sigma^{2}}{e^{2}})xM/[Cohort\;size\right]$$ (7)

Where NC–the requested sample size, Z–z-score of the confidence probability, 1.98 for α < 0.05, e–acceptable value of noise-to-signal ratio, assumed 0.1, σ—variation of the tag prevalence between random groups of the size M, M/[Cohort size]–adjustment of the variation determined in a one-time calibration for the random group of size M to various sizes of the cohorts, see [23]. The values of NC were computed for different possible outcomes with different relative variation σ and those that satisfied (7) were acceptable.

### Estimating hazard function of dying by Gompertz model using EMR records

Hazard function H(T) of dying in the year T+1 of age can be estimated empirically from the expression:

$$H(T)=(\frac{Nd}{Nc})/[LS-FPB]$$ (8)

Where N_d_ is the number of accrued decedents in the initial elderly population N_c_, entering the observation at 100% survival rate, FPB–the group-averaged year of follow up beginning, LS–the group averaged life-span.

## Results

### Data analysis flow

General flow of the data analysis is presented at Fig 1. Dementia prevalence was measured in relatively large groups of patients that took an OTC supplement or an approved pharmaceutical (Methods). These groups were produced by Pivot Table clustering of the patient’s profiles in the database, using a common dosage forms name shared by different patients. The average dementia, norm and mortality in the groups were normalized to the respective averages for the database total, forming hazard ratios. The hazard ratios were validated by clinical trials, studies and animal experiments for a given agent or related compounds which exert their action by similar mechanisms (Table 1). The agents were ranked based on a linear combination of dementia, residual cognitive norm and mortality computed in the groups, and the rank was demonstrated to correlate with clinical trial success (Tables 1 and 2). Next, the validated higher-ranking compounds (first octile of rank) were traced as combinations of N agents in the personal profiles of the patients, and the outcomes were reported (Tables 3–6, Figs 3 and 4). The role of confounding factors was ruled out and the role of the agent diversity as an independent factor was demonstrated (Table 3, Figs 3 and 4). The material of Tables 4–6 deals with permutations of the patient’s cohorts included in the studies, reproducibility of the effects in multiple independent databases, attempts to rule out biases, attempts to show that the effects persist in all ages. Supplemental files are provided for independent review of the original data and of the intermediate processed files reflecting the key elements of the data flow. Reading the text and concurrently reviewing supplemental data is the shortest path to learn the methodology. File A in S1 Data (161 MB) presents sub-files “Grouping by patient ID”, “Grouping by Drug Name”, with detailed Pivot Table templates illustrating transition from visit organization of NACC database (multiple rows with the same patient ID but different dates and content) to clusters by agent/factors or to longitudinal format (one patient ID per one row, time-dependent information presented as MIN or MAX indicating beginning and end of follow up).

### Screening of individual pharmacological mechanisms for correlation with dementia rate, preservation of cognitive norm and all-cause mortality

In NACC dataset, the columns with pharmaceuticals and supplements were identified, the agents were mapped to the patient’s visits and exposures on the first and the last visits were computed (File A in S1 Data, examples of using Pivot Tables). The 118,000 visits in the NACC database as of 5/2017 were clustered into the cohorts according to use of > 1,900 dosage forms of FDA-approved pharmaceuticals and over-the-counter supplements (see Methods, File B in S1 Data). For each group, defined by presence of an individual compound or factor, fractions of dementia, cognitive norm and all-cause mortality were compared to similar fractions in the entire database, thus producing following hazard ratios: HR DEM–hazard ratio of all-cause dementia in the category of compounds aggregated by mechanism of action, HR AMRT–hazard ratio of all-cause mortality, HR NORM–hazard ratio of the patients remaining cognitively normal (File B in S1 Data).

The groups with insufficient numbers of patients were pooled based on common pharmacological mechanism or co-use group (ex: vitamins of B group). Each mechanistically similar pooled category of the compounds (or individual compounds) was aligned with matching clinical trials (Table 1).

Additional mechanistic groups not yet tested in RCTs with neurodegeneration prevention endpoints but showing promise based on the HRNORM, HRDEM and HRAMRT signals do require further verification by clinical observations, and, therefore, are listed in Table 1 separately from the main body of compounds. For these compounds, the literature was mined for evidence of their exclusion by prescribing physicians in dementia (barbiturates, baclofen). The data presented in Table 1 suggest the inclusion of biotin, probiotics, bronchodilators, nasal steroids, chondroitin/glucosamine, and anti-gout medicines as candidate compounds to explore for their potentially neuroprotective properties (See Table 1 for references). The data on verified use of these compounds in various clinical trials formed a training set.

The question that we tried to answer was whether the signals produced based on epidemiological parameters in the database have any relevance to the reported incremental success of >250 clinical trials addressing neurodegeneration in the period 01–2007 to 10–2017, extracted in PubMed (referred to as “training set”, see Methods, File C in S1 Data). The relevance of the enquiry is driven by a known disconnect between the promising epidemiological data and incremental results in the clinical trials of individual agents [15–16].

Table 1 shows relationship between database-born metric Regression Score (REG) derived in the individual mechanisms and BAL/TOT = ((RCT+)–(RCT-))/TOT for clinical trials (Pearson correlation R = 0.35), where BAL is the balance of positive RCT+ and negative RCT- trial outcomes, TOT is the total number of trials for the mechanism of interest. The predictor was tested for stability by randomly excluding 10 (18%) of correlated pairs in the profiles of 53 pairs (details of this test are shown in File C of S1 Data).

To improve the strength of the signal, we pooled several mechanisms into one test, by computing average BAL/TOT and average REG for N pharmacological actives. Pooling was conducted in the following manner: Table 1 was ranked by REG, the top 5 agents were averaged on BAL/TOT and REG sides and the 5-member window was moved one step down the rank. As a result, a profile of 50 windows producing 5-member averages was correlated. Combining 5 agents increased correlation coefficient to 0.72 between database signals and clinical trial validations. To assess its non-randomness, the same scrambling procedure was applied producing the random arrays (see File C in S1 Data, scrambling tests). With Kolmogorov-Smirnov test p-value 0.82, Z-test followed with value of z being -5.63843. The result points to strengthening of signal-to-noise ratio at this degree of pooling and to strengthening of signal itself. Analogously pooling 10 actives in a single test led to the correlation coefficient 0.93 between the averaged predictor in the window of 10 and the averaged outcome in a window of 10 (Z-score against scrambled set = - 5.5, p-value = 3.8x10^-8).

This result has practical significance. Testing simultaneously 10 promising agents (or the number of any database-nominated protective factors that can be provided as a part of a clinical trial) is more inherently predictable and successful than testing a single agent. In a 10-member parcel, the outcome is determined by the predictor by R^2^ = (0.93)^2^ = 86%, while 14% of the future observed effect is defined by inherently unknown factors. By contrast, in a single agent test the outcome is determined by the predictor by R^2^ = (0.35)^2^ = 12%, while the remaining 88% are defined by inherent unknowns. With the outcome being trial success, the percentage of stronger biological results must theoretically increase more than linearly with the diversity (complexity) of factors included in the anti-dementia trial rationale.

Choosing highly scoring agents based on dosage form epidemiology (as dementia prevalence correlate) and using combinations of such highly scoring agents leads to greater success rate in clinical trials and clinical applications. The predictive power of database-driven metrics was tested for its ability to

1. predict success in trials based on individual mechanisms and combinations not included in the training set;
2. predict the alignment with research literature describing the effects of different pharmaceuticals on short- and long- term cognitive status.

The testing set was assembled by mining of controlled clinical trials including the top 7 agents ranked by REG (Vitamin A–lutein, chromium picolinate, biotin, selenium, zinc, antimigraine, oestrogen). Unlike training set in Table 1 identified in PubMed between 2007 and 2017 (File C in S1 Data), the testing set was extracted in Google Scholar without limitations by time. The results are combined in Table 2. In testing context, biotin and anti-migraine drugs were the agents with known high REG and no trials available in training set.

Several clinical trials were identified for biotin, including those inducing remissions in symptomatic multiple sclerosis (Tourbah et al., 2016, Table 2). Other trials of mostly nutritional multivitamin-multimineral compositions including biotin demonstrate high rate of success in the incremental definition accepted in this report (in 6 out of 8, BAL/TOT = 0.625). Strong REG signal for anti-migraine medicines was not accompanied by any tests for such in controlled trials. Instead in (Vuralli et al, 2018 in Table 2, [38], [39]), a mechanistic link between migraine and cognitive decline is discussed as under-appreciated. This leads to idea that anti-migraine medications may contribute to preventing gradual long-term cognitive decline as well, thus producing a validated database signal. The authors were not aware of the supporting literature at the time when extracting the training set was performed (Table 1). Other prominent BAL/TOT ratios were at 0.6 for carotenoids, 1.0 for chromium picolinate, 0.625 for selenium, 0.75 for zinc. For oestrogens, the scoring is complicated by apparent difference in performance of transdermal patches, non-progestin oral hormone replacement therapies and oestrogen-progestin combinations. Differentiation of oestrogen treatments into transdermal estradiol (T), generic oral treatments (E) and combinations with progesterone (P) leads to BAL/TOT = 0.84 for transdermal oestrogen (T), BAL/TOT = -0.2 for oral oestrogen (E) and BAL/TOT = 0.09 for oestrogen combined with progestin (P). Pooling all available formulations produces BAL/TOT = 0.06. The highest octile of the training set ranked by REG includes 10 incrementally positive, 3 negative and 3 neutral trials. In the testing set, the respective numbers are 45, 17 and 10. By contrast, the entire training set includes 174 incrementally positive, 104 negative and 45 neutral results, while the 2 bottom quartiles include 60 incrementally positive, 59 negative and 22 neutral results. These numbers indicate that incorporation of REG ranking in the planning of anti-dementia clinical trials is likely to produce ~ 3-fold increase in their success rate. Fig 2 presents formal Response Operative Characteristic performance data for REG classifier, with the margins of variation produced by 5:1 cross-validation, modifying the prediction rule (average of 5 re-training subsets after excluding 10 mechanisms out of the panel of 53 in each case). The permutation-averaged ratio of positives to negatives is > 5 in the highest ranking octile of the REG score, while being 0.95 for the entire dataset.

With meaning of REG rank established as an *a-priori* predictor of success rate, a total of 1962 dosage forms available in NACC were screened by computing REG and Studentized Z-scores for each group (File C in S1 Data). Relative errors for REG components (NORM, DEM, AMRT) were assessed based on a random model and adjusted to the drug group sizes after computing a propagated error when combining the components in REG, also incorporating REG variation between prediction sub-rules. Out of 1962 dosage forms, 1033 produced the groups of suitable size. Enrichment between the best octile by Z-score and the rest of the agents with the available metric produced the following leaders: vitamins D (enrichment 7, 6 df (dosage forms)), vitamins B (4.2, 8 df), vasodilators (14, 2 df), metabolic supplements (3.5, 18 df), probiotics (2.8, 7 df), antivirals (4, 13 df), stimulants (2.7, 19 df), ophthalmic cellulose (7, 2 df), omega-3 (4.2, 8 df), NSAIDs, (2.9, 27 df), magnesium (4.2, 13 df), immunomodulators (2, 32 df), hormonal non-oestrogen (2, 26 df), herbal supplements (2.4, 31 df), oestrogen (7, 12 df), antiplatelet (3.2, 6 df), antimigraine (21, 4 df), antihistamine (1.8, 33 df), anticonvulsants (2.1, 13 df), antidepressant tricyclic (1.4, 6 df). Metformin shows enrichment 21 on 4 dosage forms, but appears to be out of the trend with the entire antidiabetic pool (1.1, 23 df), with insulin itself demonstrating enrichment of 0.65 on 13 df. The effect of insulin should be put in the context of the underlying condition, which in itself favours neurodegeneration. As insulin is typically prescribed in diabetes cases with severely impacted glucose controls, effects of disease may outweigh the effects of the agent [40, 41]. Vasomotor modulators (PDE5 inhibitors) also show enrichment 14 on 3 df. On the other side, analgesics (0.45, 46 df), corticosteroids (0.34, 42 df), anti-acid (0.45, 16 df), anti-arrythmic (0.55, 13 df), antibiotics (0.5, 5 df), anti-cancer therapies (0.25, 29 df), statins (0.6, 13 df), antidepressants (0.45, 17 df), antifungals (0.4, 19 df), overall antihypertensives (0.9, 28 df), bronchodilators (0.6, 17 df) display the opposite trend of distributing in favour of the lower Z-score octiles. Thus, PDE5 inhibitors [42], antiplatelet [43, 44], vasodilators [43, 45], antimigraine [38, 39], antivirals [46], probiotics [47], magnesium [48, 49], transdermal oestrogen (Table 2) and possibly metformin [40, 41, 50, 51] are the agents of interest to perhaps combine with the multivitamin-multimineral formulas of Table 2. Conversely, the signals by corticosteroids [52, 53], anti-cancer therapies [54, 55], anti-acid drugs [56, 57], infections underlying use of antibiotics and antifungals [58], the link between dementia and respiratory deficiency [59], between dementia and cardiac arrythmia [44] mostly align with outside evidence of involvement in neurodegeneration. Effects of statins are likely due to underlying conditions outweighing the effects of medications (cardiovascular disease, metabolic syndrome), not unlike the insulin case described above. Association of anti-acids may need to be deconvoluted further in the effects of soluble aluminium and proton-pump inhibitors [57]. Overall, the profile of REG distribution coefficients between the top and the remaining octiles of REG rank for 1033 individual dosage forms agrees with literature and the observed coefficients can be rationalized through the balance between the effects of therapy and that of underlying cause on the incidence of cognitive decline.

Reviewing the clinical trial space, literature and enrichments by REG, we noted that three larger groups of active compounds can be formed:

1. Cerebrovascular modulators: antihypertensives, vasodilators, PDE5 inhibitors, antiplatelet, antimigraine.
2. Immunomodulators: antihistamine, NSAIDs, antivirals, anti-gout and anti-arthritis DMARDs.
3. Metabolic stimulators, coenzymes, antioxidants, vitamins

High correlation between averaged REG for groups > 5 and clinical trial success produces a rationale to combine these large domains in higher order compositions, and those–with the components of lifestyle [60]. Thus, we tracked the complex combinations (5–20 elements) of the factors analogous to the best performing in the clinical trial space in the personal profiles of patients across multiple databases.

### Combinatorial intervention as efficient approach to delay cognitive decline: Analysis of confounders

For each patient, we counted a total equally weighted number of the factors in highest ranking quartile by regression score REG to generate a novel index that we termed “protective complexity” which, in a sense, reflects summarized effort in staving off cognitive decline. This index can include pharmacological, behavioural, comorbidity or even social factors, as soon as they are pre-validated by controlled studies or at-least by mechanism-based outside evidence. In this report, we limit our analysis to pharmacological factors only.

Protective complexity indices PR were computed for each patient, followed by re-ranking of entire cohort by PR score, producing divisions with higher or lower protective scores. Files D-F in S1 Data illustrate protective complexity indexes and present overall layout of the computational experiments reported below.

Before detailed study of cohorts with high protective complexity index, we addressed a bigger picture of potential confounding of the effects by other factors. Table 3 and Figs 3 and 4 illustrate the contributing factors in cognitive state at the end of follow up as well as potential interferents overlapping with the effects of interest. The problem of deconvoluting these effects in the background of numerous covariates was addressed by multiple regression analysis. The confounders and the target effect were simultaneously included in the combined model and the statistical weights of the regression coefficients (t-STAT, p-value) were reported. When the outcome (Y) in the regression was cognitive status, the positive coefficients (and t-STAT values) reflect the factors that facilitate cognitive decline. The opposite is true for the negative coefficients. The protective complexity and the positive coefficient for confounders indicate co-enrichment by these covariates in the highly protected cohorts, while negative coefficients mean mutual exclusion.

Review of Table 3 (A and C) shows that pre-existing cognitive decline, age, length of follow up and prescription polypharmacy produces positive contributions in the final dementia rate in NACC and NSHAP datasets. By contrast, higher education, presence of osteoarthritis, multiple protectants, positive emotionality, good physical and mental health as baseline, and social alcohol consumption correlate with delayed cognitive decline (in NSHAP). Review of Table 3 (B and D) shows that protective complexity covariates positively with length of follow up, higher cognitive score at baseline, prescription polypharmacy, osteoarthritis, age, female gender, positive emotionality and higher education. Positive emotionality, longer follow ups and higher cognitive score at baseline themselves can be outcomes of protective complexity, but we treated these parameters as confounders and included them in multivariate background. Despite this inclusion, protective complexity produced statistically significant contributions in both NSHAP and NACC in the simultaneous presence of all expected confounding factors.

The combination of confounding factors form a background model, predicting cognitive decline rates under the assumption that the factor of interest (not included in the model) is comparably distributed between the groups, producing negligible influence on the result. The predicted and observed values of cognitive decline were equal within confidence intervals when testing the background model (Figs 3E, 4A and 4B).

This agreement with the prediction means that for the groups as large as 5–10% of the total dataset, consideration of known confounders suffices for assessing the baseline correlating with the factor of interest, even if for individual profiles the regression model and observation deviates significantly (R^2^ = 0.41 for NACC and 0.07 for NSHAP). The plots comparing the predicted baseline cognitive decline and the observed decline are in Figs 3D and 4C. In the cohorts with the highest diversity of protectants, the observed rate of cognitive decline was 3-fold lower than that predicted by confounding background. These results were outside of confidence CI95 intervals even for the smaller cohorts with the top level of protectant exposure.

Interestingly, the groups with highest cognitive score at baseline had largest intake of the protectants, possibly as a component of lifestyle. Arguably, lifestyle itself may be a key contributor to higher ***baseline*** cognitive scores, especially in the highly educated group, and in multimorbid osteoarthritic patients, in which the pain motivates to seek relief by a plethora of alternative medicine and supplementation over extended periods of time. This element of reverse causation was resolved conservatively by attributing baseline cognitive score to confounding factors and not to outcomes. If baseline cognitive score was treated as an outcome together with the final score, background model would have shown 5-fold differences vs. the observation. Thus, the confounder-corrected magnitude of the effect is in a broad range between 3-fold and 5-fold reduction as compared to the confounder-only background model.

### Combinatorial effects survive variations in the methods of data collection

True biological effects should manifest despite permutations in the method of data collection, as all objective phenomena with high signal-to-noise ratios of detection. Table 4 presents the dementia rate as a function of time in the cohorts with variable degrees of the consumption of protective compounds, as measured by PR.

In individual profiles collected in National Social Life Health and Aging Project (NSHAP), vitamins, minerals, nutraceuticals, herbals, aspirin and hormones (androgens and oestrogens) were traced as combinations. Main dataset of NSHAP, which is a self-reporting survey, is significantly depleted in dementia tags for the dataset of that size. Cognitive test results are available in NSHAP WAVE1 (2006) and WAVE2 (2011), which allow us to extract the number of errors per each component of a test, including the orientation in time, and number sequences, in a longitudinal fashion. In National Ambulatory Medical Care Survey (NAMCS), the set of profiled protective agents included angiotensin receptor blockers, bronchodilators, omega-3, vitamins D, C and multivitamins, aspirin, ibuprofen, celecoxib, as well as mineral supplement of calcium, magnesium and lithium.

Table 4 shows a strong negative correlation between the dementia fraction and the number of protective compounds reported in personal profiles in comparable age brackets. The results for dementia are paralleled by the results for stroke, the latter being a precursor and correlate of dementia. Notably, in groups with higher number of protective compounds (PR), the deferral of dementia onset was matched by proportional decrease in all-cause mortality. For each dataset, the dementia rates were converted into hazard ratios measured as [most protected]/[least protected], The Pearson correlation between the number of protective mechanisms (protective complexity) present in the person’s profile and hazard ratio for dementia as well as for all-cause mortality were -0.91 (P <5.6x10^-5) and -0.92 (P < 1.1x10^-4), respectively (See File E in S1 Data, negative correlation between cognitive decline outcomes and protective complexity). Observed increases in lifespan (LS) are consistent with blocking of dementia—one of several major fatal conditions. In the cohorts ranked according to the number of protective compounds (PR), a 4-fold decrease in dementia burden between the top 2% and bottom 49% of 72–73 years old, and a 2.5-fold decrease of 78–79 years old strata were detected. The size of this effect decreases at ages > 90 but to approximately 1.4-fold (NACC2 data).

In both the NSHAP and NACC datasets, the rates of cognitive decline accumulation were dependent on the number of protective mechanisms (PR) covered per patient. In the NAMCS dataset which allowed extraction of the combinations of supplements, angiotensin receptor blockers, bronchodilators and COX2 inhibitors, trends in dementia reduction between more and less protected cohorts were stronger than in the NSHAP dataset, where only supplements, salicylates and hormones were profiled.

To minimize possibility that the trends presented in Table 4 are due to poor balancing of confounders between the cohorts, the distributions of potential confounding factors between Top 5% and Bottom 49% was equalized (Methods) for NACC. The values are provided in the Table 4 addendum. In Tables 5 and 6 below, the equalization of confounders between the cohorts produced by the same equalization protocol was confirmed statistically by p-value of T-test.

The results observed for protective complexity are applicable to a broad spectrum of neurological diseases. Specifically, 3-fold differences were observed for frontotemporal dementia and Parkinson’s disease tags, 2.5-fold for seizures, and a ~1.5–fold for vitamin B12 deficiency and chronic alcohol dependence; all these parameters behaved as a function of protective complexity (PR). It is worthwhile to note, however, that the protective effects correlating with high PR were not limited to neurodegeneration. In the NSHAP and NAMCS data, the hazard ratio for chronic renal disease in the most protected/least protected cohorts was 0.3–0.5 after normalization by the total comorbidity loads. We concluded that the effects of this report are stable across the methods of producing a dataset and methods of confounder normalization.

### Combinatorial effects are more effective when applied in cognitively normal state then in mild cognitive impairment (MCI)

**Fig 5:**
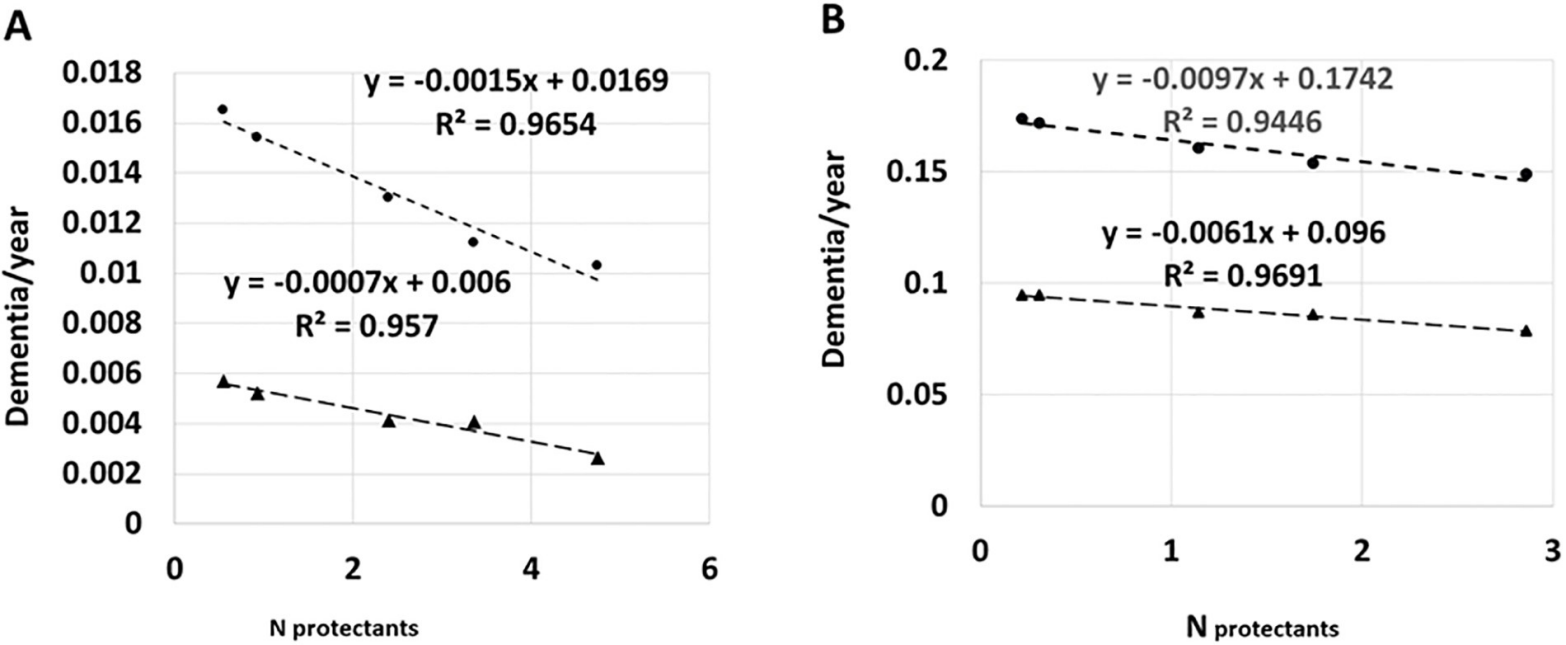
Annual rates of conversion to dementia (fraction increment/year) from a cognitively normal state (Fig 5A) or MCI (Fig 5B) as a function of protective complexity (PR) in the patient’s profiles.

To ensure that baseline dementia tags are excluded from groups of patients consuming certain groups of active compounds, all NACC records labelled with any dementia-related tags, including mild cognitive impairment (MCI) or pre-MCI were removed, and the rest of patient profiles (N = 13,355) were analysed longitudinally by accounting only for emergent dementia related tags. Fig 5A presents the kinetics of the conversion from a cognitively normal state to dementia in cognitively normal cohorts stratified by number of potentially protective compounds consumed. Separately, analysis was performed in cohort diagnosed with MCI at inception (N = 7,350). Fig 5B presents kinetics of the conversion to dementia in the records of MCI group.

The circles indicate the dementia levels accumulated at the end of follow-up, and the triangles indicate time-averaged values of dementia accumulation.

DEMav.=DEMEx(TE-TD)/(TE-TB)

where DEM av. is the time-averaged dementia; DEM E is the final accrued dementia value at the end of follow-up; (TE–TD) is the interval of time between the beginning of clinical dementia at TD and the end of follow-up TE; and (TE–TB) is the interval of time between the beginning TB and the end TE of follow-up.

In Fig 5A, the follow-up interval is 3.5 years; in Fig 5B, the follow-up interval is 2.5 years. The cohorts are normalized by age, gender, education, comorbidity, and unrelated polypharmacy.

The results shown in Fig 5A and 5B indicate that in groups exposed to higher protective complexity the rates of the transition to dementia slow down, and dementia onset is later in the more protected stratum. This conclusion follows comparison of final and time-averaged dementia rates born in the originally cognitively normal population. On the other hand, the rates of the conversion to dementia among patients in pre-existing MCI were higher than that in cognitively normal participants, in agreement with the previous reports [54] and were less amenable to delay by the combinations of protectants.

### The effects of protective complexity impact all stages of aging and are life-long

In the next step, we tested if the protective effects are observed in decedents. (Files G, H, I, J in S1 Data). Using the fraction of mortality with dementia diagnosis in the total mortality, we minimize the biases associated with different place of different patient sub-sets along aging trajectory, with some groups closer to terminal decline and some more healthy. To minimize possible population heterogeneity bias even further, we restricted analysis to a newer release of NACC (09–2018, Version 3), confining to a single version 3.

Comparison of Tables 4 and 5 data points to qualitative reproducibility of all effects despite use of different NACC versions and years of release. In the presence of multiple protectants (PR > 5), the accrued dementia, rate of dementia accrual, rate of MMSE decline and mortality hazard function are reduced, while the length of follow up and life expectancy are increased with significant p-values vs low protectant control. In addition to Table 4, the Tables 5 and 6 demonstrate that the effects described in this article are not transient but persist through the entire lifespan as visible in the diminished dementia rate in decedents. No major confounders such as age, education, gender ratio, arthritis, diabetes, cardiovascular events, sum of comorbidities and polypharmacy were statistically different to explain the observation. Both equalization and multiple regression approaches to confounder neutralization produce comparable results.

Angiotensin-receptor blockers (ARBs) were demonstrating high enrichment by REG in the leading rank octile. The consistent interest in these agents as anti-dementia protectants [61] motivated us to include ARBs in the compositions together with [Zn, Se, Cr, biotin, vitamin-A/lutein, herbals] sub-set (leading octile by REG), to amplify the number of mechanistic domains putatively countering dementia. Any of these agents were considered with equal and traced in the patient’s personal profile producing the count of different species (protective complexity). The profiles were re-ranked by protective complexity and the results of the study are presented in Table 6. The Table 6 demonstrates a deeper negative correlation with dementia rate (0.04 at the age 70 and 0.14 at the age 75 in a more protected group vs. 0.21 and 0.31 for the same 5-year difference in the control in a living population; 0.066 at the age 74 and 0.266 at 81.5 in a more protected group vs. 0.51 at the age 75 and 0.76 at 80.6 in a control for decedent category). The T-test statistic is provided in the Table 6. This reduction in dementia frequency is observed in different permutations of the exposures in decedents (File G in S1 Data) and follows high REG for the group with [Zn, Se, Cr, biotin, vitamin-A/lutein, herbals], also confirmed by observed performance (BAL/TOT).

The addendum of Table 6 presents the test of multicomponent compositions in 9203 decedents for all versions of NACC together (Files I, J in S1 Data). We tested the decedent subsets receiving compositions of comparable complexity, of comparable proportion for prescription and OTC components, with close levels of confounders–but with different average REG and Z-score of the component groups. If non-compliance of cognitively impaired patients is the driving factor for exclusion of pharmaceuticals from personal profile data–this setting provides a test to the hypothesis of retrospective bias as a source of observed dementia declines in EMR. Both higher and lower REG groups maintain complex scheduling with similar proportions of OTC components and the group with the higher average REG/Z-score is expected to demonstrate greater life-long dementia reduction.

Both compositions 1 (14 complexity elements in the regimen) and 2 (13 complexity elements in the regimen) require comparable scheduling and disruption of such scheduling by cognitive decline as a cause of observation is unlikely. High exposure to the protective compounds was measured within 1.5–2 years before dying, with the MMSE cognitive scores off the maximum value. The background model in Figs 3D and 4C show that the drift in confounder composition of Table 6 as a function of protective complexity gradient can lead to substantial differences in confounder-induced baseline, but not enough to explain the entire effect. The pattern of this dataset is more consistent with deferral of dementia onset and progression as a function of REG/ Z-score.

## Discussion

To identify potential therapeutics suitable for the prevention of dementia or the slowing down of age-associated cognitive impairment, we have conducted a meta-analysis of the longitudinal patients’ cohorts with the records presented in three datasets: NAMCS, NSHAP and NACC, totalling about 50000 patients. These results point to an inverse relation between the number of top-ranking active agents present in a patient’s or respondent’s profile and the fraction of dementia in such groups. Uncovered relationships are stable as they reproduce in partial subsets of the same database, across age ranges and across different and independently processed databases (Tables 3–6). Our findings support observations made in the reports of Bredesen, who produced the conclusions similar to ours [62]. Amounts of agents in a profile were also reciprocal to the rate of mortality accrual and positively correlated to lifespan (Tables 3–6, Supplemental materials). Confounder analysis by multiple regression and factor-by-factor equalization of major confounders confirmed independence of the confounder-adjusted effects attributable to the diversity of protective compounds.

Before proceeding further, we should note that electronic medical record (EMR) studies are correlative in nature. While causal inference is made from experimental evidence, EMRs should be utilized as a guide for efficient designing of this experimentation. Likewise, we should treat putatively beneficial combinations of protective compounds as a “composite marker” or a “signature” of neuroprotection phenotype, not as proven mechanistic mediator of neuroprotection as such. Utilizing databases as sole source of data may generate systematic reverse-causation biases, for example, due to a tendency of cognitively impaired individuals to neglect healthy lifestyle or adhere to pharmaceuticals or supplements. While we rigorously tried to address this problem (Figs 3 and 4, S1 Data, Tables 3–6), complete negation of this bias is difficult to produce. While controlling for known confounders and sources of possible errors, a bias due to yet unknown confounders may still be present. For example, certain co-morbidities evidenced by additional polypharmacy may compete with dementia as a potential cause of death, leading to a decrease in dementia rates. Indeed, given [q’] as an inherent probability of dementia, the probability [q] of non-dementia causes decreases the effective q” = (1-q) x [q’], irrespective of mechanistic interaction between the diseases. Accumulation of competing co-morbidities correlates with accumulation of complexity in the patient’s profile. We attempted to take this bias into account by equalizing the number of comorbidities as well as prescription polypharmacy through conducting factor-by-factor confounder equalization. We report that observed effects persisted in factor-by-factor normalized datasets as well (Table 4).

In view of these limitations, we explored evidence collected in controlled clinical trials by decreasing the threshold of what is considered “a success” in such trials. Following minimal definition such as “confirmation of original hypothesis”, many clinical trials yield positive results by showing incremental improvement of cognitive scores or delay of cognitive score decline or delay of other symptoms of neurodegeneration. If epidemiological signals in EMR databases arise due to protective agents as such, and not by underlying Bayesian competitive factors, EMR-based findings should correlate to the incremental success of the treatments with same agents detected in controlled trials.

Indeed, higher ranking candidates in NACC database demonstrated non-random correlation between measured quantitative signals and success of clinical trials, exploring these agents. This correlation reaches > 0.9 for larger groups of agents (10 or more factors per profile) and such groups are readily available in general population, producing an element of evidence that dementia can be controlled by such approaches (in both age of onset and in the extent of prevalence). These conclusions were produced based on the analysis of > 50000 patients, > 1900 dosage forms and > 300 clinical trials linked in a single alignment presented earlier (Tables 1 and 2). The mechanisms at the highest tier of database signals in Table 1 and Files A-C in S1 Data (Mg+2, Zn+2, Se, biotin, chromium picolinate, vitamin A-lutein) are traditionally prescribed as life-extending or preventing retinal degeneration and substantial evidence of mechanistic involvement or clinical efficiency are available for each, including successful controlled clinical trials or successful treatment of organic neurological conditions in humans, based on incremental definition of “success” ([63–72], Tables 1 and 2).

Perhaps the strongest argument in favour of feasibility of database-driven anti-dementia therapy design is the existence of MEND protocol, currently tested in 100 patients (2018) [62], up from the original 10 first reported in 2014 [73]. In this approach, the factors identified in databases and literature as dementia modifying are all combined in a single multi-modal intervention, tailored to an individual phenotype and the list of necessary pharmaceuticals. MEND is found to reverse cognitive decline progressing up to MCI stage [73] and increasing cognitive scores by 5–7 units or more. A significant cognitive decline delay was observed in a multidomain lifestyle FINGER trial (2-year multidomain intervention including diet, exercise, cognitive training, vascular risk monitoring, [74]), conducted by an independent group and in magnesium trial [75]. The results align with large-scale earlier observations of reversing cognitive declines [76]. Theoretically, the results of [72–75] can be explained by a combination of high random MMSE variation in year-to-year tests and multiple comparison contexts, when multiple research groups stage Phase I anti-dementia trials worldwide. But our data in > 50000 patients indicate strong negative correlation between complex combinations of factors analogous to those in [72–75] and dementia prevalence. This argues against insufficient trial sizes in [69–75] as the reason for the observations, plus the results can be pooled as produced by similar multi-component methodologies. Specifically, MEND involves multiple subgroups recruited in the study over multiple years. The main table in [73] illustrates a consistent record in the same direction of improvement of both cognitive score and imaging data. This consistency over 4 years (2014–2018) is difficult to explain away by a study size alone, especially in the context of our findings.

Perhaps in our data the elements of MEND arise non-intentionally due to antihypertensive, vasodilator, antiplatelet, antidiabetic, antihistamine and NSAID polypharmacy combined with use of supplements and covariate components of healthy lifestyle. As a minimum, these life-long, non-transient >3-fold decreases of dementia risk suggested by our study deserve surveys of smaller subsets of patients identified by protective complexity signatures. These surveys (clinician assessment, psychometric analysis) could identify additional correlates of benign polypharmacy presence that may lead to deliberate application of these factors in controlled therapeutic setting. The possibilities of compounded placebo effects and Hawthorne effect in the treated vs control population control need to be revisited as well [77].

Regardless of the actual mechanism, our study clearly indicates that, in elderly populations, multi-modal supplementation engaging multiple neuroprotective pathways correlates with sizeable delays in cognitive impairment and conversion to dementia. Importantly, these effects are “dose-dependent” in a sense that higher degrees of protection are provided by larger functional variety of the consumed compounds rather than an increase in utilized amounts of any single compound (Table 1–6, Figs 3–5). Notably, in individuals already staged as having MCI, engaging protective complexity regimens (or lifestyle correlates of such regimes) appear to be less efficient in preventing further sliding towards dementia than in cognitively intact cohorts (Fig 5). These results are consistent with relative lack of clinical success in trials conducted in individuals already experiencing significant cognitive impairment [78, 79].

The magnitude of the maximal observed effects identified by the signatures of the report is significant and can be tracked in Table 6, second part. Among the individuals that become decedents within 3 to 7-year observation range, the most protected stratum demonstrated 9.7% presence of dementia at 79 years age (FPB, baseline) and in the least protected stratum (control) the percentage was 63% in 76 years old patients (baseline). After reaching 85 years (FPE), the most protected patients demonstrated 31% of dementia within 1–2 years before dying. By contrast, in the control group, 76% of the patients at the average age of 78.7 years (FPE) were diagnosed with dementia within 1–2 years before dying. Assuming doubling of dementia prevalence each 5 years in this range [3], age-adjusted hazard ratio becomes ~ 0.1 at the age of 76 and ~ 0.2 at the age of 80. Minimization of dementia prevalence is consistent with > 6 years of increase in lifespan.

Present analysis does not include detailed investigations of other factors which were curiously influential in decreasing dementia prevalence. Decreases of the odds to develop dementia in presence of osteoarthritis are analysed in detail elsewhere [80]. It is likely that an investigation of the full space of the factors defining the risk of dementia will lead to other significant findings. For example, unexpected reduction of dementia rates was reported in the study of anti-viral combinations for controlling Herpes Zoster; these finding were made by tracing prescription signatures in more than 28,000 patients in Taiwan [46].

To summarize, our large-scale retrospective study of more than 50,000 patients extracted from 3 independent EMR sources (NACC, NSHAP, NAMHC) had explored more than 1,900 OTC supplements and FDA-approved dosage forms and their combinations for their potential to prevent dementia. Strongest negative correlations with diagnosed dementia and/or dementia progression rate were identified for herbals, Zn, Se, Mg, biotin, vitamin A, lutein and chromium picolinate. In context of EMR, the supplements are often reported in combinations. These combinations correlate with cognitive outcomes much better than any single supplement under other equal conditions (Table 1 and narrative analysis). Results of our study may aid the design and the interpretation of the data collected in frame of clinical trials. In short, our study presents the results of systematic exploration of combinatorial preventive treatment regimens for age-associated multi-morbidity, with an emphasis on neurodegeneration, and provides compelling evidence for their feasibility.

## Supporting information

S1 Table List of downloadable datasets and intermediate analytic files deposited at https://dataverse.harvard.edu and at https://data.mendeley.com/datasets/. (DOCX)

S1 Data Files A-J. (DOCX)

S1 Checklist (DOC)
