# Supplementary material for: Pharmacological signatures of the reduced incidence and the progression of cognitive decline in ageing populations suggest the protective role of beneficial polypharmacy
Source: PLoS One. 2019 Nov 6;14(11):e0224315. doi: 10.1371/journal.pone.0224315 (PMC6834256; doi:10.1371/journal.pone.0224315)
Supplement: S1 Table — (DOCX) [file pone.0224315.s001.docx]

**Supplemental data for the manuscript** *“****Pharmacological signatures of the reduced incidence and the progression of cognitive decline in ageing populations suggest the protective role of beneficial polypharmacy”***

**By Dr. Anatoly Mayburd, Mathilda Koivogui and Prof. Ancha Baranova**

The datasets supporting this study are deposited at <https://dataverse.harvard.edu> and at <https://data.mendeley.com/datasets/>.

**The links are tabulated.**

**Important note: many EXCEL files are bulky and in order to download them properly we recommend “Save as” step to be followed by “Open” step. Using the immediate “Open” option may cause a delay**.

| **Dataset** | **URL** | **Description** |
| --- | --- | --- |
| Supplemental File S1 | <https://doi.org/10.7910/DVN/R8Z0TT> | NACC data in visit form, original file as provided by NACC, release as of 9/2018, with pharmaceuticals mapped on the visit space. |
| Supplemental File S2 | <https://doi.org/10.7910/DVN/OR2XGH> | >1900 clusters formed in Pivot Tables based on drug names. |
| Supplemental File S3 | <https://doi.org/10.7910/DVN/RQREHT> | Clinical trial space is aligned with database signals producing REG predictor of clinical trial success for a pharmacological mechanism. |
| Supplemental File S4A | <https://doi.org/10.7910/DVN/0SWLJW> | crude effects, non-adjusted for confounders |
| Supplemental File S4B | <https://doi.org/10.7910/DVN/EHWMQT> | Supports Table 3, Figures 3 and 4, multiple regression analysis, NSHAP results |
| Supplemental File S4C | <https://doi.org/10.7910/DVN/JEVLOE> | Supports Table 3, Figures 3 and 4, multiple regression analysis, NACC results |
| Supplemental File S5A | <https://doi.org/10.7910/DVN/2UODXU> | Support for Tables 5 and 6 of the Main Manuscript. |
| Supplemental File S6A | <https://doi.org/10.7910/DVN/YZBI5Z> | Describes experiment with limiting final MMSE to > 25. Shows that the trends are preserved in this regime, addressing reverse causation bias. Indeed, the average MMSE in the case and control is above the level of dementia and therefore is unlikely to impact adherence to the prescriptions. |
| Supplemental File S6B | <https://doi.org/10.7910/DVN/YZBI5Z> | This file compares two regimes with close level of complexity. The regimes differ in the average REG, but include close numbers of components. The experiment rules out the role of adherence to the regime as origin of effects, instead emphasizing the role of REG. Addresses reverse causation bias. |
| NACC database in the original form with the drugs mapped (2017 release) | doi:10.17632/bg8mpdmkdz.1;  <https://data.mendeley.com/datasets/bg8mpdmkdz/1> | NACC dataset is downloaded as of 05/2017 and the agents of interest are mapped on the profiles of the patients. |
| NACC Code Book. | Harvard dataverse: <https://doi.org/10.7910/DVN/GMBGYH>;  Mendeley.com:  <https://data.mendeley.com/datasets/x5v529kzmf/1> | NACC Code Book, allows to interpret the dataset. |
| NACC in longitudinal format. | Mendeley.com:  doi:10.17632/zfzgdm3gx7.1;  <https://data.mendeley.com/datasets/zfzgdm3gx7/1> | NACC dataset was converted in longitudinal form, the values of parameters at the beginning and at the end of follow up are provided |
| Risk factors | doi:10.17632/f468f9p555.1;  <https://data.mendeley.com/datasets/f468f9p555/1> | This file provides references reporting hazard ratios of dementia in different medical conditions. |
| NSHAP dataset, combined WAVE1 2005-2006 and WAVE2 2010-2011 | doi:10.17632/4fkpvb4g35.1; <https://data.mendeley.com/datasets/4fkpvb4g35/1> | The NSHAP survey was performed longitudinally as a set of waves. The exposures to the pharmaceuticals were mapped to the patient’s profiles and this allowed to measure the rate of dementia progression, as well as cognitive decline reversions. |
| NSHAP WAVE 1 and WAVE 2 Code Books | Harvard dataverse:  <https://doi.org/10.7910/DVN/GMBGYH>;  Mendeley.com:  doi:10.17632/ngtd8y8nw7.1;  for 2010-2011 Code Book; <https://data.mendeley.com/datasets/ngtd8y8nw7/1>;  doi:10.17632/6yv87yppcp.1; for 2005-2006 Code Book;  <https://data.mendeley.com/datasets/6yv87yppcp/1> | Code Books are provided to interpret NSHAP data. |
| National Ambulatory Medical Care Survey (NAMCS) | doi:10.17632/psmcz23j37.2; <https://data.mendeley.com/datasets/psmcz23j37/1> | The drugs and diagnoses are encoded in this dataset. The drugs are encoded in NAMCS database (<https://www2.cdc.gov/drugs/applicationnav1.asp>) , the Drug Codes are provided in the dataset. Diagnoses are provided in ICD-9 notation. After decoding, the pharmaceuticals and diagnoses were mapped on the personal profiles of ambulatory patients. |
| National Ambulatory Medical Care Survey (NAMCS) Code Book | Harvard dataverse:  <https://doi.org/10.7910/DVN/GMBGYH>;  Mendeley.com:  doi:10.17632/n88cxr9nhg.1; <https://data.mendeley.com/datasets/n88cxr9nhg/1> | Code Book is provided to interpret NAMCS data. |
