# Supplementary material for: Pharmacological signatures of the reduced incidence and the progression of cognitive decline in ageing populations suggest the protective role of beneficial polypharmacy
Source: PLoS One. 2019 Nov 6;14(11):e0224315. doi: 10.1371/journal.pone.0224315 (PMC6834256; doi:10.1371/journal.pone.0224315)
Supplement: S1 Checklist — (DOC) [file pone.0224315.s003.doc]

| **Section/topic** | **#** | **Checklist item** | **Reported on page #** |
| --- | --- | --- | --- |
| **TITLE:** Pharmacological signatures of the reduced incidence and the progression of cognitive decline in ageing populations suggest the protective role of beneficial polypharmacy | | |  |
| Title | 1 | Meta-analysis |  |
| **ABSTRACT** | | |  |
| Structured summary | 2 | Background objective: Preventive treatments for dementia are warranted. Here we show that utilization of certain combinations of prescription medications and supplements correlates with reduced rates of cognitive decline.  Data sources: NACC, NSHAP, NAMCS  Study eligibility criteria: All members of the retrospective datasets  Participants and interventions: Elderly individuals were compared based on the number of supplements and herbals per person  Study appraisal and synthesis method: More than 1,900 FDA-approved agents and supplements were collapsed into 53 mechanism-based groups and traced in electronic medical records (EMRs) for >50,000 patients. These mechanistic groups were aligned with the data presented in more than 300 clinical trials, then regression model was built to fit the signals from EMRs to clinical trial performance. While EMR signals of each single agents correlated with clinical performance relatively weakly, the signals produced by combinations of active compounds were highly correlated with the clinical trial performance (R = 0.93, p = 3.8 x10^-8). Higher ranking pharmacological modalities were traced in patient profiles as their combinations, producing protective complexity estimates reflecting degrees of exposure to beneficial polypharmacy.  Results: For each age strata, the higher was the protective complexity score, the lower was the prevalence of dementia, with maximized life-long effects for the highest regression score /diversity compositions. The connection was less strong in individuals already diagnosed with cognitive impairment. Confounder analysis confirmed an independent effect of protective complexity in multivariate context. A sub-cohort with lifelong odds of dementia decreased > 5-folds was identified; this sub-cohort should be studied in further details, including controlled clinical trials.  Limitations: The study is retrospective and the origin of the effects requires additional studies, Unknown confounders cannot be ruled out a-priori.  Conclusions and implications of the study: In short, our study systematically explored combinatorial preventive treatment regimens for age-associated multi-morbidity, with an emphasis on neurodegeneration, and provided extensive evidence for their feasibility. |  |
| INTRODUCTION | | |  |
| Rationale | 3 | Clinical trials of dementia preventing interventions are mostly successful, considering multiple supplements and factors of lifestyle. We tested a hypothesis that combining preventive factors decreases dementia in inverse proportion to the number of the factors. |  |
| Objectives | 4 | The study addresses dementia at the end of follow up as a function of the number of protectants in the person’s profile. The patients in the lowest half of the patient’s rank by the combined factor exposure are the control, while the highest octile or 2% of the rank is the group of interest. |  |
| METHODS | | |  |
| Protocol and registration | 5 | NA. |  |
| Eligibility criteria | 6 | All patients in the database, irrespective to the follow up or any other factors were considered eligible. |  |
| Information sources | 7 | Published or freely available datasets produced by NACC, NSHAP, NAMCS; also Pubmed and Google Scholar for literature information. |  |
| Search | 8 | PubMed was searched first producing training set. Keywords “cognitive decline”, “dementia”, “Alzheimer’s disease”. Combined with “clinical trial”. For testing set, the same search was conducted in Google Scholar. |  |
| Study selection | 9 | Publications were included in meta-analysis if they mentioned randomized clinical trials in the context of dementia prevention. Incremental success was defined as the agreement with the initial hypothesis. |  |
| Data collection process | 10 | Abstracts of the publications as well as the tables of the compositions were explored for hazard ratios of dementia prevention, for the opinions of the trial organisers if the original hypothesis is confirmed and what supplements were included in the trial. |  |
| Data items | 11 | Incremental success of clinical trials, defined as + 1 if the original hypothesis is confirmed by the trial, -1 if not confirmed and 0 is inconclusive. These metrics are aligned with the supplement compositions tested in the trials. |  |
| Risk of bias in individual studies | 12 | Placebo controlled and randomized trials were the source of data. The more biased data (clinical studies, retrospective studies, animal models) were considered only if clinical trials were absent for the supplement. They were not included in quantitative data for the meta-analysis. |  |
| Summary measures | 13 | The meta-analysis measures correlation coefficients between the incremental success for agents that follow clinical trials and hazard ratios of dementia reduction matching the same agents in the databases.  Across the table of promising supplements, the correlation is measured between two columns of numbers. The first column are the results of clinical trials, the second column are the matching database-born hazard ratios. |  |
| Synthesis of results | 14 | See 13. The data for multiple supplements are synthesized, allowing to align database signals and clinical trial results across multiple factors. |  |

Page 1 of 2

| Section/topic | # |  | Reported on page # |
| --- | --- | --- | --- |
| Risk of bias across studies | 15 | The bias is minimal on modern clinical trial side. Placebo control and normalization are the conditions for selection of the trials in the analysis. |  |
| Additional analyses | 16 | Predictive metrics based on database signals and directed to forecast the clinical trial results |  |
| RESULTS | | |  |
| Study selection | 17 | >250 clinical trials addressing >1900 individual pharmacological dosage forms for the OTC supplements and herbals formed the training set. > 75 clinical trials were included in the testing set. |  |
| Study characteristics | 18 | PUBMED references were provided for each data point and the original data can be reviewed independently. |  |
| Risk of bias within studies | 19 | Minimal |  |
| Results of individual studies | 20 | + 1 – benefit within original hypothesis, -1 – absence of benefits, 0 – inconclusive. |  |
| Synthesis of results | 21 | Correlation between clinical trials and database signals for a profile of 55 pharmacological mechanisms. The correlation coefficients were assessed for randomness by Kolmogorov statistical test. |  |
| Risk of bias across studies | 22 | The studies were selected based on simple criteria and without any discrimination. |  |
| Additional analysis | 23 | Figure 2 relates enrichment in successful clinical trials to the rank of database signals. |  |
| DISCUSSION | | |  |
| Summary of evidence | 24 | The main result of the meta-analysis is realization that retrospective database signals are predictive of the clinical trials, if they belong to the top octile level of the ranked database signals. |  |
| Limitations | 25 | Incomplete retrieval was considered. Two sources – such as PubMed as well as Google Scholar were applied for cross-verification. |  |
| Conclusions | 26 | Alignment of >250 clinical trial results in the training set and > 70 clinical trial results in the testing set with the signals produced by retrospective databases of electronic medical records validated the latter as a predictor of meaningful biological effects at the trial level. Especially strong correlations were measured when the parcels of agents were present in a profile of the person.  The methods open the path to combinational therapy design. . |  |
| **FUNDING** | | |  |
| Funding | 27 | NA |  |

*From:*  Moher D, Liberati A, Tetzlaff J, Altman DG, The PRISMA Group (2009). Preferred Reporting Items for Systematic Reviews and Meta-Analyses: The PRISMA Statement. PLoS Med 6(7): e1000097. doi:10.1371/journal.pmed1000097

For more information, visit: **www.prisma-statement.org**.

Page 2 of 2
